# Comprehensive routine diagnostic screening to identify predictive mutations, gene amplifications, and microsatellite instability in FFPE tumor material

**DOI:** 10.1186/s12885-020-06785-6

**Published:** 2020-04-07

**Authors:** Elisabeth M. P. Steeghs, Leonie I. Kroeze, Bastiaan B. J. Tops, Leon C. van Kempen, Arja ter Elst, Annemiek W. M. Kastner-van Raaij, Sandra J. B. Hendriks-Cornelissen, Mandy J. W. Hermsen, Erik A. M. Jansen, Petra M. Nederlof, Ed Schuuring, Marjolijn J. L. Ligtenberg, Astrid Eijkelenboom

**Affiliations:** 1 Department of Pathology, Radboud university medical center, PO Box 9101, 6500 HB Nijmegen, the Netherlands; 2 Department of Pathology, Princess Máxima Center for Pediatric Oncology, Utrecht, The Netherlands; 3 Department of Pathology, University Medical Center Groningen, University of Groningen, Groningen, The Netherlands; 4 Department of Human Genetics, Radboud university medical center, Nijmegen, The Netherlands; 5 Department of Pathology, The Netherlands Cancer Institute, Amsterdam, The Netherlands

**Keywords:** Predictive analysis, Next-generation sequencing, Mutation, Gene amplification, Microsatellite instability, FFPE, Melanoma, GIST, Colorectal carcinoma, Lung cancer

## Abstract

**Background:**

Sensitive and reliable molecular diagnostics is needed to guide therapeutic decisions for cancer patients. Although less material becomes available for testing, genetic markers are rapidly expanding. Simultaneous detection of predictive markers, including mutations, gene amplifications and MSI, will save valuable material, time and costs.

**Methods:**

Using a single-molecule molecular inversion probe (smMIP)-based targeted next-generation sequencing (NGS) approach, we developed an NGS panel allowing detection of predictive mutations in 33 genes, gene amplifications of 13 genes and microsatellite instability (MSI) by the evaluation of 55 microsatellite markers. The panel was designed to target all clinically relevant single and multiple nucleotide mutations in routinely available lung cancer, colorectal cancer, melanoma, and gastro-intestinal stromal tumor samples, but is useful for a broader set of tumor types.

**Results:**

The smMIP-based NGS panel was successfully validated and cut-off values were established for reliable gene amplification analysis (i.e. relative coverage ≥3) and MSI detection (≥30% unstable loci). After validation, 728 routine diagnostic tumor samples including a broad range of tumor types were sequenced with sufficient sensitivity (2.4% drop-out), including samples with low DNA input (< 10 ng; 88% successful), low tumor purity (5–10%; 77% successful), and cytological material (90% successful). 75% of these tumor samples showed ≥1 (likely) pathogenic mutation, including targetable mutations (e.g. *EGFR*, *BRAF*, *MET*, *ERBB2, KIT, PDGFRA*). Amplifications were observed in 5.5% of the samples, comprising clinically relevant amplifications (e.g. *MET, ERBB2, FGFR1)*. 1.5% of the tumor samples were classified as MSI-high, including both MSI-prone and non-MSI-prone tumors.

**Conclusions:**

We developed a comprehensive workflow for predictive analysis of diagnostic tumor samples. The smMIP-based NGS analysis was shown suitable for limited amounts of histological and cytological material. As smMIP technology allows easy adaptation of panels, this approach can comply with the rapidly expanding molecular markers.

## Background

Personalized medicine becomes increasingly important in cancer treatment. Drugs targeting specific mutated genes or specific activated pathways are clinically available for several indications, e.g. for *EGFR*-mutated lung cancer [1]. Currently, many more targeted therapies are being evaluated in clinical trials and show promising results either alone or in combination with other drugs [2]. As molecular markers are constantly expanding, predictive analysis should be easily adaptable to future clinical need. In this era of personalized medicine, next generation sequencing (NGS) analysis using gene panels is increasingly becoming a standard diagnostic approach as interrogation of multiple molecular markers is required with only a limited amount of (mostly) formalin-fixed, paraffin-embedded (FFPE) tissue [3]. These markers include a spectrum of genetic alterations, ranging from small base pair alterations (e.g. point mutations, small deletions, or small insertions) to larger structural variants (e.g. translocations, amplifications, or deletions) affecting genes or large regions of chromosomes. Besides therapy decision, the presence or absence of specific genetic alterations can contribute to the differential diagnosis and provide relevant prognostic and predictive value [4]. In addition, mismatch repair deficiency, causing instability of repetitive DNA sequences known as microsatellites, was shown to be predictive for response to immune checkpoint blockade for a range of tumor types [5].

Sequencing is considered a high resolution approach for the detection of small genetic alterations. With the introduction of sensitive NGS techniques, mutations can reliably be detected in a very low number of cells. We have previously implemented and validated the single molecule Molecular Inversion Probes (smMIP) approach that includes molecular tagging to identify PCR duplicates. These duplicate reads are merged into consensus reads, which leads to the elimination of PCR and sequencing artifacts. In combination with the strand-specific analysis to distinguish genuine mutations from deamination artefacts, it is possible to detect point mutations, small deletions, small insertions, and complex mutations (indels) down to at least 1% mutant allele frequency, as well as to reliably exclude sequence variants with a mutant allele frequency > 3% [6]. In addition, existing gene panels are easily adaptable [6] and thereby this technology can comply with the growing clinical need. Taken together, the smMIPs technology allows screening of multiple therapeutic targets with high sensitivity using FFPE tissue material in a routine diagnostic setting.

Detection of copy-number variations (CNVs) at specific genomic locations, e.g. amplification of *MET*, is frequently performed by fluorescence in situ hybridization (FISH). In addition, several genome-wide approaches are available, including (shallow) whole genome sequencing or (SNP) array. Interestingly, in the last few years it was shown that NGS-based approaches using defined gene panels are able to identify CNVs in parallel to sequence alterations [7–10]. Recommendations were published to guide the detection and reporting of copy number gains using gene-panel NGS data in a routine diagnostic setting [11].

Recent developments, showing clinical benefit of immune checkpoint blockade in microsatellite instability (MSI) positive tumors, increased the importance of tumor agnostic MSI detection [5]. MSI is characterized by spontaneous gains or losses of nucleotides in small nucleotide repeat regions (microsatellites) and reflects a state of genomic hypermutability. In diagnostic FFPE tissue, the microsatellite status is routinely investigated by a pentaplex PCR investigating five microsatellites [12]. This analysis was mainly validated in Lynch syndrome associated tumors, but little data is available regarding the reliability of this analysis in other tumor types [13, 14]. Studies focusing on the spectrum of unstable microsatellites across multiple cancers show different patterns of MSI among cancer types [15–17]. NGS analyses can be used to study multiple microsatellite loci in one test [13, 17–21].

Taken together, different molecular techniques (i.e. NGS, FISH, and pentaplex PCR) are often used for predictive analyses for cancer therapy. NGS gene panel analysis allows detection of these genomic aberrations in one assay, which will potentially save valuable time, material and costs. Therefore in the current study we aimed to develop a single smMIP-based NGS assay, which can detect sequence alterations, amplifications, and MSI. To comply with the increased number of therapeutic opportunities based on molecular aberrations, both in regular therapeutics and in clinical trial settings, our panel was designed to allow predictive screening for the four most requested molecular-screened indications, i.e. lung cancer, colorectal cancer, melanoma and gastro-intestinal stromal tumors (GIST), but is also useful for evaluation of a broad spectrum of other tumor types as well. Molecular markers for initial therapy decisions as well as markers important for disease progression (i.e. resistance mechanisms) were included in the panel. In addition, we validated and implemented the detection of clinically relevant gene amplifications in compliance with recently published recommendations [11], and we included the analysis of 55 microsatellites for MSI detection across multiple cancer types.

## Methods

### Panel design

The Predictive Analysis for THerapy (PATH) panel should cover all clinically relevant predictive markers for molecular testing of lung cancer, colorectal cancer, melanoma, and GIST. To select predictive markers that have a consequence for targeted treatment-decision-making, clinicians and pharma companies were consulted, as well as the scientific societies (e.g. Dutch Association of Chest Physicians (NVALT), melanoma, and GIST-Consortium), and literature and clinical trial registries were screened for relevant genomic alterations for the specified cancer types. A combination of occurrence, predictive value, and clinical consequences guided the selection of a sequencing panel encompassing 13 kb (Table 1, Supplementary Table 1). Consensus of the panel was obtained with molecular biologists of three specialized hospitals.

**Table 1:** PATH panel

Mutation analysis	Amplification analysis	MSI
AKT1	ALK	55 MSI markers
AKT2	BRAF	
AKT3	EGFR	
ALK	ERBB2	
ARAF	FGFR1	
BRAF	FGFR2	
DDR2	FGFR3	
EGFR	KIT	
ERBB2	KRAS	
FGFR1	MDM2	
FGFR2	MET	
FGFR3	PDGFRA	
GNA11	PIK3CA	
GNAQ		
GNAS		
HRAS		
IDH1		
IDH2		
JAK2		
KIT		
KRAS		
MAP2K1		
MDM2		
MET		
MTOR		
NRAS		
PDGFRA		
PIK3CA		
POLE		
PTEN		
RAF1		
ROS1		
TP53		

### Sample preparation

Samples were prepared as previously described [6]. In short, gDNA was isolated from FFPE tissue sections (generally 3 × 20 μm) using 5% Chelex-100 and 400 μg proteinase K followed by purification using NaAc and EtOH precipitation. DNA concentrations were measured using the Qubit Broad Range kit (Q32853; ThermoFisher, Waltham, MA). Previously, we reported correlations between a low DNA concentration, the number of years between tissue sampling and DNA isolation, and suboptimal smMIP-based NGS results [6]. However, no clear thresholds could be defined. As pre-sequencing analyses are time-consuming, which hampers the turnaround time of the majority of samples, the smMIP-based NGS analysis was performed on all samples without preselection based on DNA quality.

### Preparation of the smMIP pool

smMIPs for novel targets were designed using the MIP pipeline as described previously [6, 22]. In brief, for mutational analyses preferentially each region had to be covered by two independent smMIPs targeting both strands (double tiling). Hotspots including surrounding regions or complete exons (including splice sites) in 29 genes were selected for mutational analyses. For gene amplifications at least 10 smMIPs per gene were used, which were spread throughout the gene without double tiling. For some genes the smMIPs that were designed for mutation detection fulfilled these criteria, whereas for other genes additional smMIPs were designed (Supplementary Table 1–2). Seven smMIPs for amplification analysis showed inefficient capture and sequencing of targeted regions. Therefore these smMIPS were replaced by newly designed smMIPs on adjusted target regions (Supplementary Table 2). To provide a sex control, smMIPs targeting X-chromosomal AMELX and Y-chromosomal AMELY were added to the panel. During smMIP design the presence of common SNPs (minor allele frequency > 0.2%) under probe sequences was avoided. If unavoidable, smMIPs were designed to recognize the variant alleles, resulting in 76 additional smMIPs. For MSI detection, smMIPs targeting the five microsatellites of the pentaplex PCR analysis [23] were included, resulting in a total of 554 smMIPs. In a later stage, smMIPs for 50 additional microsatellite regions were designed and added to the sequencing panel (Supplementary Table 3–4).

The designed smMIPs (Integrated DNA Technologies (IDT), Leuven, Belgium) were pooled in an equimolar fashion. The smMIP pool was phosphorylated with 1 μL of T4 polynucleotide kinase (M0201; New England Biolabs, Ipswich, MA) per 25 μL of 100 μmol/L smMIPs in ATP-containing T4 DNA ligase buffer (B0202; New England Biolabs, Ipswich, MA). Based on the obtained coverage for each smMIP, the pool was rebalanced by increasing the concentration of some of the smMIPs. The molecular ratio between gDNA and smMIPs was set to 1:3200 based on 100 ng gDNA input.

### Automated smMIP library preparation and sequencing

Library preparation was performed as previously described [24]. The final purified libraries were denatured and diluted to a concentration of 1.2 pmol/L. Sequencing was performed on a NextSeq 500 (Illumina, San Diego, CA) according to the manufacturer’s instructions (300 cycles Mid-Output or High-Output sequencing kit), resulting in 2 × 150 bp paired-end reads.

### Variant detection

Data analysis was performed as previously described [6, 24]. The first steps of data conversion were done automatically, i.e. BCL-to-FASTQ conversion and demultiplexing [24]. Demultiplexed FASTQ files were uploaded and analyzed in Sequence pilot version 4.4.0 (JSI medical systems, Ettenheim, Germany) [6], which included removal of *PIK3CA* and *PTEN* pseudogene reads from the alignment and subsequent analysis. After variant calling, all variants were manually inspected and curated. To improve detection of large deletions resulting in skipping of *MET* exon 14, we decreased the percentage of consecutive bases that have to match to the reference without a mismatch (30% instead of 50%) and the minimum total absolute coverage for both directions combined (10 instead of 20). To evaluate detection of large deletions resulting in skipping of *MET* exon 14, three artificial DNA sequences (IDT) were used.

### Amplification analysis

An external baseline control series was generated from ten normal FFPE tissue samples, which were sequenced in several independent runs. The unique coverage per tumor sample was normalized using the median sequencing depth of all amplicons in the sample, an approach adapted from Budczies et al [7]. This normalized coverage per gene per sample was divided by the mean coverage of the gene in the control series. The obtained value includes the relative unique coverage (or fold change) and can be divided by two to assess the total number of alleles that are present per genome equivalent. In addition, a significance score, the z-score, was calculated by dividing the difference between the normalized coverage of the sample and the control series by the standard deviation of the control series. To assess the number of alleles that are present in the tumor cells only, the relative coverage was corrected by tumor purity: (relative coverage * 2 – (1 – fraction of neoplastic cells)*2) / fraction of neoplastic cells [11].

### Microsatellite instability detection

mSINGS software was used for the detection of significantly altered read length distribution in sequencing reads covering microsatellite markers, as described by Salipante et al [20]. This tool is able to perform tumor-only MSI analysis without the need of a paired normal sample. In short, baseline reference values were generated from 20 normal FFPE tissue samples for the 55 microsatellite loci. Microsatellite status of unknown samples was assessed by comparing repeat length distribution for each locus to the baseline reference value. Per locus, the total number of alleles with different lengths was assessed. Repeat lengths were included if their read count exceeded 5% compared to the read count of the most frequently observed allele. The number of repeat lengths was compared to the number of the baseline. If the counted repeat lengths exceeded [mean number of alleles + (2 x SD)] the baseline value, a locus was scored as unstable. Finally, the mSINGS score was assessed by dividing the number of unstable loci per samples by the total number of evaluated loci.

### OncoScan CNV array

80 ng DNA from FFPE samples was processed according to the manufacturer’s instructions (ThermoFisher Cat. No. 902694). Data was visualized and analyzed using Chromosome Analysis Suite (ChAS) software version 3.2 (ThermoFisher).

### Panel requirements

The requirements of the PATH panel were formulated prior to its validation and implementation, including i) > 90% of selected regions of interest should be double tiled, preferably recognizing both strands for hotspot regions. ii) Read depth distribution throughout the panel should be uniform; the average read depth for > 90% of the targets relevant for sequencing analysis may vary up to 1 order of magnitude. iii) The number of sample drop-outs should be similar or smaller compared to the smMIPs cancer hotspot panel [6] and concordant results should be obtained. iv) For amplification analysis, 5 positive controls and 10 samples without any amplification should be correctly classified. v) For MSI detection, a series of 10 positive and 20 negative control samples should be correctly annotated.

## Results

### Panel validation – sequencing analysis

**Fig. 1:**
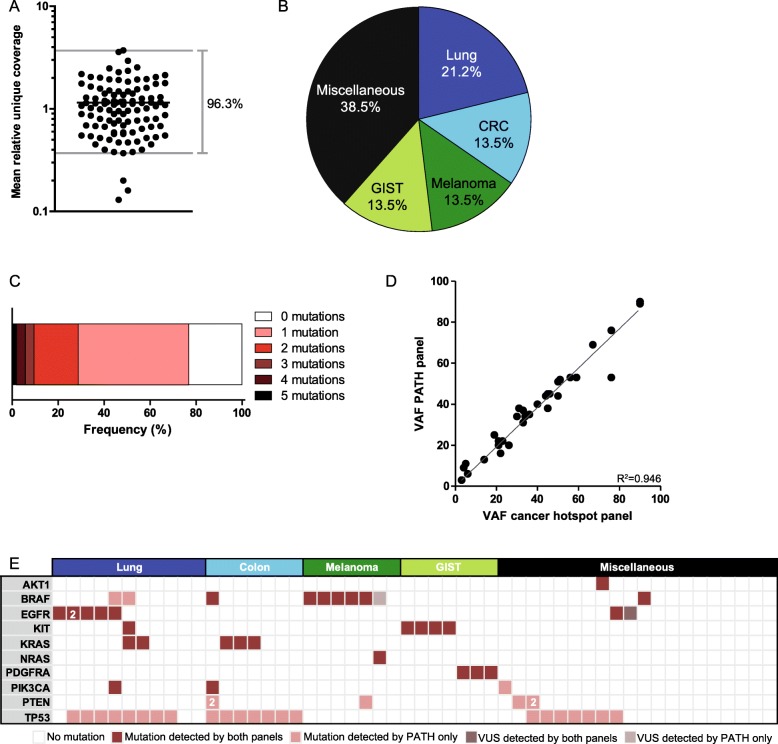
Validation of sequencing analysis. **a** Average read depth of 96.3% of the targets is within one order of magnitude. For every region (hotspot or whole exon) the mean coverage was calculated per sample. By dividing the mean coverage per region through the median coverage in a specific sample, the mean relative coverage for each region was defined. The average of the mean relative coverage per region of 10 samples is plotted. **b** 57 samples distributed over the indications lung, colon, melanoma, GIST, and miscellaneous (analysis in the context of evaluating clinical trial options) were sequenced and compared with the current routine diagnostics panel. **c** Percentage of samples with a specific number of mutations. **d** The variant allele frequencies (VAF) of the 32 mutations identified by both the PATH panel and the cancer hotspot panel. **e** Mutations detected in the 52 samples successfully sequenced during the validation phase. Dark red indicates mutations identified with both panels (cancer hotspot panel and PATH panel). Light red indicates additional mutations identified using the PATH panel. ‘2’ indicates two different mutations identified in the same gene. Dark and light purple indicates variants of unknown significance (VUS) identified by both panels or PATH panel only, respectively

The smMIPs were pooled in an equimolar fashion and tested on 4 samples of archived FFPE tumor tissue. To boost coverage of underperforming smMIPs relevant for sequence analysis, the concentration of 55 smMIPs was increased (Supplementary Table 2). In addition, 7 smMIPs were replaced by newly designed smMIPs. 10 samples from archived tissue were sequenced using this rebalanced pool. 91% of relevant regions for mutation detection were covered by ≥2 smMIPs in both orientations. Unique read depth obtained from sequence analysis of these 10 samples resulted in a median coverage of 506 unique reads per region, representing an equal number of individually sequenced gDNA molecules. On average, unique coverage of 96.3% of relevant regions was within one order of magnitude (Fig. 1a). The coverage of 3 regions was lagging behind, i.e. the regions around *ARAF* codon 214, *ERBB2* exon 18, and *ERBB2* exon 21. These regions are relatively GC-rich (GC% *ARAF* region: 62%, *ERBB2* exon 18: 66%, *ERBB2* exon 21: 59%), which might partly explain why these regions are more difficult to amplify and sequence (Supplementary Table 5), as previously shown [6]. Since these regions are not frequently mutated, we accepted the lower coverage of these regions.

Subsequently, 57 tumor samples (NSCLC, colorectal carcinoma, melanoma, GIST, and a range of tumor types for which clinical trial options were evaluated (hereafter referred to as ‘miscellaneous’) were sequenced using this new pool (Fig. 1b) and compared with our routine diagnostics cancer hotspot panel [6]. Parallel analysis showed a comparable number of drop-outs (*n* = 5) and a comparable coverage for the individual regions (Supplementary Fig. 1). The 5 drop-out samples could be explained by poor DNA quality, i.e. four FFPE samples were stored for > 8 years and for one sample a cytological specimen with partly degraded cells was used. Noteworthy, another five FFPE samples stored for > 8 years performed well. 75% (39/52) of the successfully evaluated cases harbored ≥1 (potentially) pathogenic mutation (Fig. 1c). 32 mutations were identified by both panels and showed concordant variant allele frequencies (Fig. 1d). The PATH panel identified 31 additional (potentially) pathogenic mutations, which were located in regions that were not present in the cancer hotspot panel (Fig. 1**e**).

In addition, we evaluated the detection of *MET* exon 14 skipping mutations, which can either be point mutations, deletions, or indel mutations that target the splice donor or acceptor site of exon 14, or the intronic polypyrimidine tract region [25–27]. In the Hs746T cell line, a c.3082 + 1G > T mutation at the splice donor site of *MET* (NM_001127500.2), predicted to result in *MET* exon 14 skipping, was successfully detected by the PATH panel. The panel design includes smMIPs with large insert size specifically designed to allow amplification of large deletions at this locus. Three artificial DNA sequences containing a 46, 173, or 201 bp deletion affecting the splice acceptor site (based on Frampton et al [25]) were detected, suggesting that large deletions affecting one of the splice sites could be detected by the panel in a diagnostic setting. Indeed, after implementation in routine diagnostics, a splice donor site mutation was identified in *MET* intron 14 (c.3082 + 1G > A) as well as a 66 bp deletion (c.2942-4_3003del) involving the *MET* exon 14 splice acceptor site. Based on these results and the different *MET* aberrations described [25, 26], we estimated that all point mutations and > 90% of all deletions that can result in exon 14 skipping can be identified by gDNA sequencing using the PATH panel.

### Panel validation – amplification analysis

**Fig. 2:**
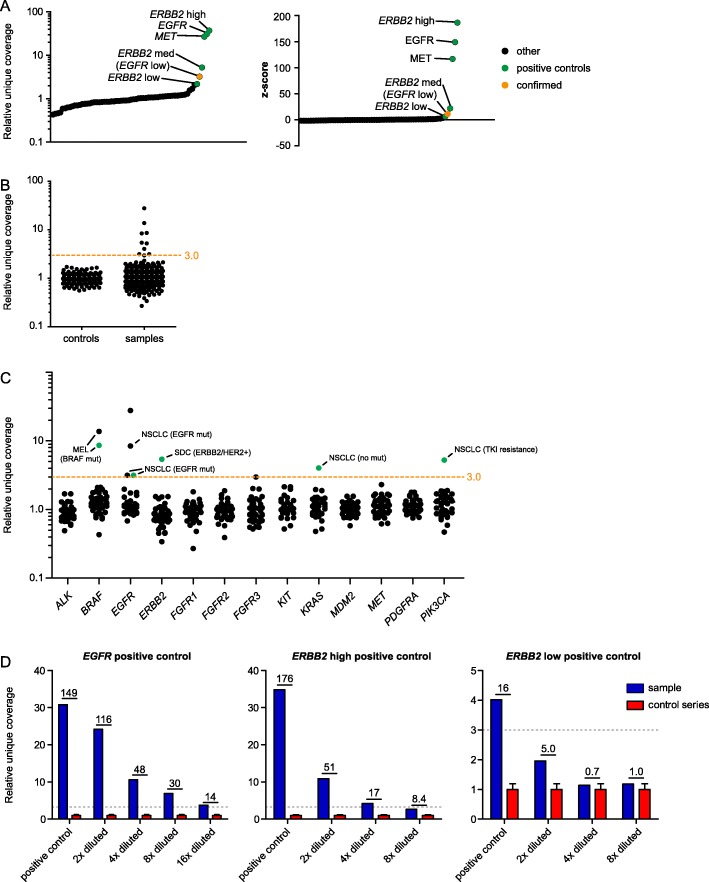
Validation of detection of amplifications in smMIP-based NGS analysis on gDNA from clinical FFPE specimens. **a** Relative coverage and z-scores in 5 positive controls for *EGFR, MET* and *ERBB2* (*n* = 3: high, medium, and low level amplification) normalized to a normal tissue control series. Values of all 13 genes relevant for amplification detection (see Supplementary Table 1) are plotted for the five samples. Values were calculated per gene per sample and sorted by increasing value. The positive control values (one gene per samples) are depicted in green, values for all other genes (12 per samples) are shown in black. The additional detected *EGFR* amplification, shown in orange, was confirmed by FISH. **b** Grouped relative coverage in a series of 46 clinical tissue samples and 15 normal tissue controls. Values were calculated per gene per sample. **c** Relative coverage per gene in the series of 46 clinical samples, with additional clinical/molecular information (details in main text). The cut-off for validation (relative coverage ≥3.0) is shown by an orange line. Potential amplifications in green were validated by OncoScan array analysis (the others were not analyzed by OncoScan array). **d** Three positive control samples were diluted in gDNA isolated from normal tissue. Relative coverage (y-axis) and z-scores (above bars) compared with a normal tissue control series are shown. On the x-axis the dilution based on gDNA concentration is shown. *EGFR* positive control: unknown tumor purity. *ERBB2* high positive control: 70% tumor cells. *ERBB2* low positive control: 50% tumor cells

An external baseline control series was generated from ten normal FFPE tissue samples, with stable performance in multiple runs (Supplementary Fig. 2A). The efficacy of amplification analysis was evaluated in five positive control samples, which included a high level amplified *EGFR*, *MET*, and *ERBB2* (HER2) sample, and a median and low level amplified *ERBB2* sample. For sample normalization, median sequencing depth over all amplicons was used, as this is much less affected by high level amplifications compared to the mean and summed unique coverage (Supplementary Fig. 2B). All amplifications in the positive controls were confirmed, as visualized by a high relative coverage and z-score compared to the other genes in the samples (Fig. 2a). In addition, the values for the *ERBB2* amplified samples were proportional to the results obtained by *ERBB2* FISH analysis, yielding a ‘high’, ‘median’ and ‘low’ value. Moreover, an additional *EGFR* amplification was observed in one *ERBB2* positive control, which was confirmed by FISH analysis (not shown).

For reliable detection of amplifications in routine diagnostics, it is highly recommended to establish cut-offs for amplification detection [11]. The relative coverage of 46 samples from routine diagnostics were therefore compared to a control series (Fig. 2b, Supplementary Fig. 3A). Based on visual inspection, a threshold in relative coverage ≥3.0 was chosen for further validation, which corresponded to a z-score of > 6.4 (Supplementary Fig. 3B). In addition, the lower limit of alleles that need to be present in the neoplastic cell component to reach the threshold of relative coverage ≥3.0 can be deduced based on the tumor purity of a sample (Supplementary Fig. 3C) [11]. Nine of 46 samples exceeded the threshold for one or more genes (Fig. 2c). The observed amplifications complied with clinical specifications and additional molecular findings [28–33]: we detected an *EGFR* amplification in four samples (i.e. three *EGFR*-mutated lung tumors and one glioblastoma specimen), a *BRAF* amplification in two samples (both *BRAF*-mutated melanoma samples), a *PIK3CA* amplification in one sample (*EGFR-*mutated NSCLC case that showed progression upon tyrosine kinase inhibitor treatment), a *KRAS* amplification in one sample (NSCLC case without any identified mutations), and an *ERBB2* (HER2) amplification in one sample (salivary duct carcinoma). Amplifications in five samples were confirmed by OncoScan array analysis (Fig. 2c (green marker), Supplementary Fig. 4). Copy number gains with a relative coverage between 2.0 and 3.0 are marked as ‘potentially amplified’ to allow follow-up analysis in case the gene amplification is clinically relevant for that particular case.

Cross-validation of the amplification analysis and the threshold in relative coverage was performed on a series of samples containing amplifications of *FGFR1, KRAS, MDM2, MET*, or *PDGFRA* (Supplementary Table 6). The amplifications that were not detected by the PATH panel analysis were below the limit of detection due to a low tumor purity, or subclonal event as determined by FISH (Supplementary Table 6). A dilution series of three positive controls diluted in gDNA isolated from normal tissue further demonstrated the importance of both tumor purity and level of amplification for the sensitivity of the smMIP-based NGS analysis (Fig. 2d**,** Supplementary Table 6). High level amplified *EGFR* and *ERBB2* samples were detected up to 16 and 4 times dilution, respectively. However, the *ERBB2* amplification in the median amplified sample was only detectable in the undiluted sample. Taken together, limitations in sensitivity of the smMIP-based NGS analysis in samples with a low tumor purity and/or low level amplifications need to be taken into account. To specify these limitations, the number of required gene copies that can be reliably detected by the smMIP-based NGS analysis given the tumor purity should be documented.

Finally, the effect of the number of analyzed gDNA molecules on the amplification analysis was studied. In two diluted normal tissue specimens, the amount of gDNA did not affect the relative coverage of the 13 genes (Supplementary Fig. 5A). In addition, samples from the validation series were grouped in low (median amplicon coverage < 25 gDNA molecules) or high (≥25 gDNA molecules) coverage (i.e. 25 gDNA molecules is set as lower limit of detection for variant analysis [6]). The group with the low unique coverage showed more outliers (Supplementary Fig. 5B), which is most likely due to technical variation due to suboptimal gDNA quality and warrants careful consideration to prevent false positive calls.

In conclusion, the PATH panel can be used to detect gene amplifications, which are considered reliable if they have a relative coverage ≥3, an amplified signal clearly above the noise based on visual inspection of all genes and in all duplicate or triplicate analyses, and if the median unique coverage per amplicon is > 25 unique gDNA molecules.

### Panel validation - MSI detection

**Fig. 3:**
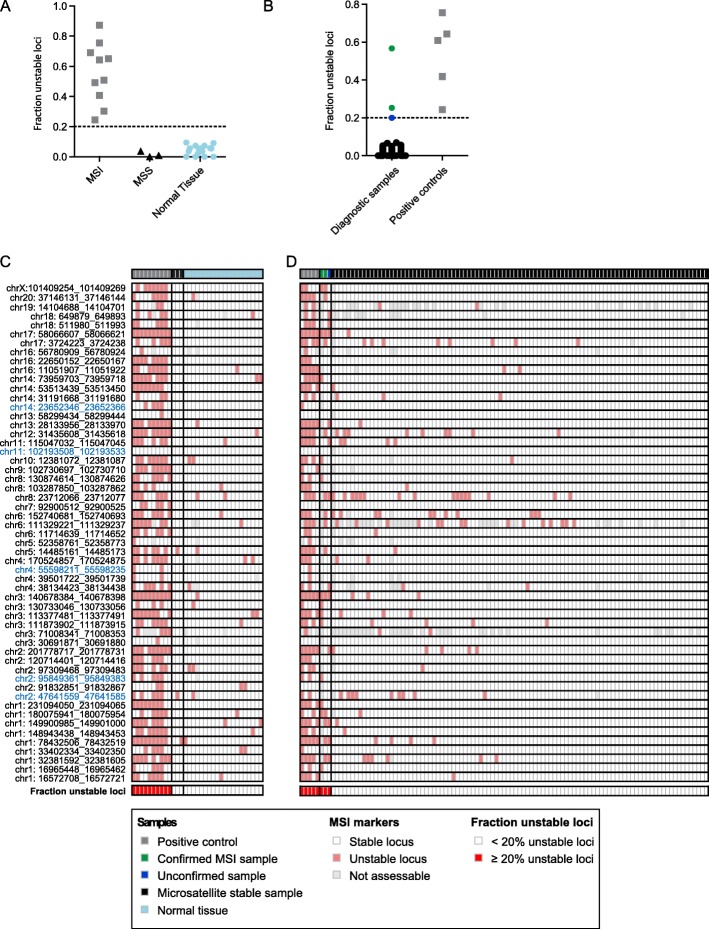
Validation of MSI detection in smMIP-based NGS analysis on gDNA from clinical FFPE specimens. **a** The fraction of microsatellite loci that showed an MSI event is depicted for MSI tumor samples, MSS tumor samples, and normal tissue samples. **b** The fraction of microsatellite loci that showed an MSI event is depicted for 100 diagnostic samples and 5 positive control samples. **c** Landscape of MSI events in the different microsatellite loci of the samples that are shown in panel A. Each column represent a tumor sample. Each row represents a microsatellite locus. Colored (red) bars represent unstable loci, white bars represent microsatellite stable loci, and grey bars represent microsatellite loci which could not be analyzed due to poor quality. The top row shows which sample is depicted: positive control (grey), microsatellite stable tumor sample (black), or a normal tissue sample (light blue). The bottoms row indicates whether the fraction of unstable loci exceeds 20% (red) or is below 20% (white). On the left of the figure the location of the microsatellite loci is depicted. Loci that are shown in blue represent the pentaplex PCR markers. **d** Landscape of MSI events in the different microsatellite loci of the samples that are shown in panel B. Each column represent a tumor sample. Each row represents a microsatellite locus. Colored (red) bars represent unstable loci, white bars represent stable loci, and a grey bar means that the locus could not be analyzed. The top row shows which sample is depicted: positive control (grey), an MSI sample that is confirmed by another technique (green), a potential MSI sample that could not be confirmed by another technique (blue), or microsatellite stable diagnostic sample (black). The bottoms row indicates whether the fraction of unstable loci exceeds 20% (red) or is below 20% (white)

For MSI analyses, a total of 63 microsatellite repeats were selected that were recurrently unstable in a range of MSI unstable tumor types [15, 16]. After initial testing, 55 markers passed the minimal requirements, as also specified by the mSINGS software. To determine the sensitivity of the markers, we analyzed the microsatellite status of 20 normal tissue samples, 3 microsatellite stable (MSS) tumor samples, and 10 MSI-high tumor samples (i.e. colorectal, endometrial, prostate, and salivary duct carcinomas) as assessed by Genescan analysis of a pentaplex PCR [23] and/or immunohistochemistry (IHC) of the mismatch repair genes. mSINGS software detected a significantly different read length in 22–87% of microsatellite markers for the MSI-high samples. Normal samples and MSS tumor samples showed 0–9% unstable loci (Fig. 3a, c). When applying the previously published cut-off of 20% unstable loci, a 100% sensitivity and specificity was reached [20]. The sample that scored the lowest fraction of unstable loci (22 and 27% in duplicate analysis) was an *MSH6* deficient prostate tumor, which showed a more subtle shift in the pentaplex PCR analysis compared to other positive controls (Supplementary Fig. 6). In three positive control samples (two endometrial tumors and the MSH6-deficient prostate carcinoma) none of the microsatellites included in the pentaplex PCR assay was classified as unstable by the smMIP-based NGS analysis, suggesting the sensitivity for these individual loci is lower than the pentaplex PCR due to the objective cut-off (mean number of alleles + (2 x SD)). Nevertheless, the cut-off of 20% unstable loci was also reached in these three samples due to instability of other loci, demonstrating the strength of multiple microsatellite makers.

The stability of the assay was examined by the evaluation of 5 MSI-high positive controls (2 colorectal carcinoma, 2 endometrial carcinoma, and 1 prostate cancer sample) in two independent sequencing runs. Reproducible results were obtained (Supplementary Fig. 7A). To challenge the threshold of 20% unstable loci, 3 positive control samples were diluted to mimic decreasing tumor load. Sample 1 harbored a high number of unstable loci and hence MSI was detected up to a neoplastic cell purity of 12.5% (Supplementary Fig. 7B). However, the fraction of unstable loci was lower in sample 2 and 3 and consequently MSI could not be detected below 35 and 30% neoplastic cell purity, respectively. These results show the importance of both the percentage of tumor cells and the fraction of unstable loci for the sensitivity of the MSI analysis. Based on these results, we decided to use a cut-off of ≥30% neoplastic cell purity to reliably report an MSS results.

Finally, the MSI analysis was evaluated in 100 samples for routine diagnostics. Addition of MSI smMIPs to the PATH panel resulted in near identical coverage of all targeted regions (Supplementary Fig. 7C) and did not affect the above described variant and amplification detection (data not shown). Besides the positive controls, three diagnostic samples (tumor purity > 80%) exceeded the threshold of 20% unstable loci (56, 25, 20%), which all three appeared to be lung cancer samples (Fig. 3b, d). IHC (loss of MLH1 and PMS2) and pentaplex PCR analysis confirmed MSI in the sample with 56% unstable loci (Supplementary Fig. 7D). In the sample with 25% unstable loci (25 and 24% in duplicate analysis) subtle changes were observed in the PCR pentaplex analysis. No material was available for IHC analysis. For the third sample (19 and 21% unstable loci in duplicate analysis), none of the PCR pentaplex markers showed instability, which was in agreement with stability of these same loci in the smMIP-based NGS analysis. The IHC analysis did not generate conclusive results for PMS2 and MLH1. However, the markers for MSI evaluation by IHC and PCR pentaplex analysis were validated on Lynch syndrome associated tumors. Little data is available regarding the reliability of the IHC and PCR pentaplex analysis in lung cancer [14, 17, 20, 34–37]. Therefore, it remains elusive whether this sample should be considered as MSI-high.

Taken together, these observations prompted us to use a threshold of 30% unstable microsatellites to classify a sample as MSI-high. Samples that show < 15% unstable microsatellite loci are classified as MSS. For samples with 15–30% unstable microsatellites IHC of mismatch repair proteins and pentaplex PCR is used to clarify the MSI status.

### Analysis in routine diagnostics

As all formulated requirements of the PATH panel were realized, the PATH panel (Table 1) was implemented as routine diagnostic procedure for predictive analyses for cancer therapy within the Radboudumc. In 2018, 745 routinely available diagnostic samples (35% lung cancer, 17% melanoma, 8% colorectal carcinoma, 3% GIST, and 37% miscellaneous tumors (a range of tumor types that is evaluated for clinical trial options)) were sequenced for variant detection and gene amplification (Supplementary Table 7). These samples were either analyzed in the context of first line treatment selection or detection of therapy resistance. MSI markers were added in a later stage and therefore MSI analysis was performed only in a subset of these samples (*n* = 478). In 18 tumor samples sequencing analysis was not successful due to low quantity or poor quality of the DNA (2.4% sample dropout; Supplementary Table 7–8). In addition, MSI could not be excluded by the smMIP panel in 53 samples due to too low tumor purity (< 30%). 728 routine diagnostic tumor samples were sequenced with sufficient sensitivity (i.e. sufficient reads for reliable variant detection [11]), including samples with a low amount of DNA (< 10 ng; 28/32 samples successful), low tumor purity (5–10%; 10/13 samples successful), and cytological samples (56/62 samples successful). Both DNA quantity and quality influence the quality of the sequence analysis (Supplementary Table 8) and consequently, the minimum required DNA input amount will vary per sample. Therefore, no minimal input requirements were determined and all samples were subjected to smMIP-based NGS analysis.

**Fig. 4:**
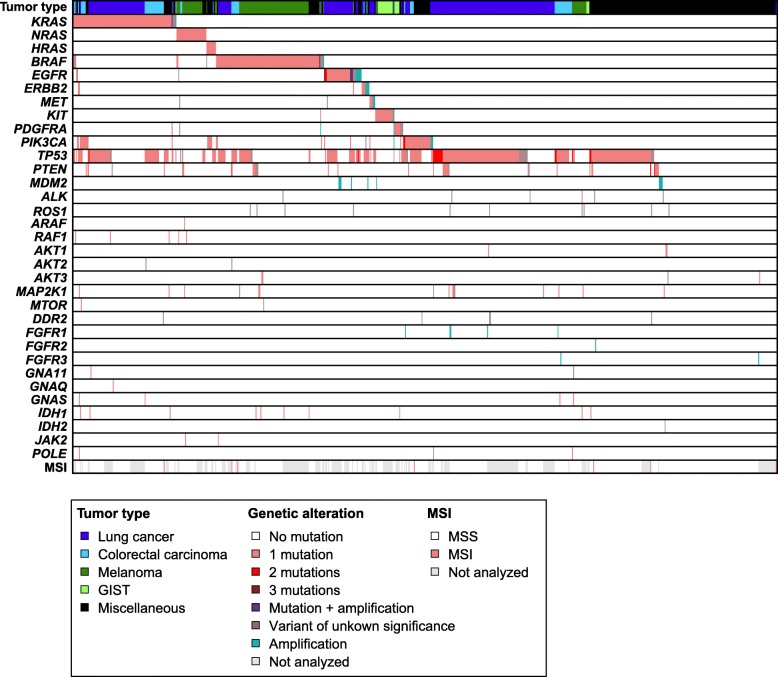
Mutational landscape of 729 tumor samples, which were analyzed with the PATH panel in routine diagnostics. (Likely) pathogenic mutations, variants of unknown significance, amplifications, and MSI status are depicted in the figure. Tumor samples are sorted on genetic alterations, followed by tumor type, and MSI status. Each column represents a tumor sample. Each row represents a genetic alterations (i.e. mutation (light red, red, or dark red), CNV (cyan), or MSI (light red). A colored bar represents a genetic alterations, a white bar represents no alteration, and a grey bar represents not analyzed. The top row shows the tumor types of the analyzed samples: lung cancer (dark blue), colorectal cancer (light blue), melanoma (dark green), GIST (light green), or miscellaneous (black)

The PATH panel showed its capability to detect splice site mutations (e.g. *MET*: c.3082 + 1G > A), large deletions (e.g. *MET*:c.2942-4_3003del), large insertions (e.g. *KIT*: c.1721_1768dup), and complex deletion-insertion mutations (e.g. *KIT*: c.1924_1936delinsGAAGTCCTGAGTC; Supplementary Table 7). In total, 788 (likely) pathogenic mutations, and 94 variants of unknown significance were detected. Variant allele frequencies ranged from 2 to 100%. 75% (547/728) of the patients harbored ≥1 (likely) pathogenic mutations, including genes suitable for targeted treatment options (e.g. *EGFR*, *BRAF*, *MET*, *ERBB2, KIT, PDGFRA*). Mutations in *KRAS, NRAS, HRAS, BRAF, EGFR, ERBB2, MET, KIT,* and *PDGFRA* were mostly mutually exclusive (Fig. 4, Supplementary Fig. 8). Mutations occurred in tumor types as expected: *KRAS* mutations were mainly observed in colorectal carcinoma (43%) [38] and lung cancer (22%) [1]; melanoma patients showed a high frequency of *BRAF* (56%) and *NRAS* (20%) mutations [39–41]; *EGFR* (12%)*, ERBB2* (1%)*,* and *MET* (1%) mutations could be mainly attributed to lung cancer samples [1]; almost all samples suspected of GIST showed a mutation (91%) in *KIT* (68%) or *PDGFRA* (23%) [42]. In the remaining tumor types, 57% of the samples harbored a (likely) pathogenic mutation and/or amplification, including amplifications of *ERBB2* and *EGFR*, and mutations in *BRAF* and *POLE*. In general, *TP53* was the most frequently mutated gene: 1 or 2 *TP53* mutations were observed in 46% (334/728) of the tumor samples [43].

Amplification analysis showed 44 gene amplifications in 5.5% (40/728) of the evaluated samples (Supplementary Table 7). Amplification of *MDM2* was detected most frequently (1.4%; 3 lung cancer, 1 melanoma, 2 liposarcoma, 2 urothelial carcinoma samples), which is reported to be associated with hyperprogressive disease after immunotherapy treatment [44]. Hence, this finding might contribute to the therapy decision. In addition, potential targetable amplifications were detected, i.e. high level amplifications of *ERBB2* [45–47] (colorectal carcinoma, salivary duct carcinoma, prostate cancer, ovarian cancer), *MET* [48, 49] (lung cancer, esophageal carcinoma)*,* and *FGFR1* [50] (lung cancer, colorectal carcinoma).

The PATH panel detected MSI in 7 of the 478 (1.4%) analyzed tumor samples, including three MSI-prone tumors (i.e. 2 of 50 colorectal cancer, 1 of 6 endometrial cancer), and four non-MSI-prone tumors (i.e. 1 lung cancer, 1 prostate cancer, 1 salivary duct carcinoma, and 1 tumor of unknown primary origin; Supplementary Table 7). The number of unstable loci ranged from 41 to 76% and thereby all these tumors were classified as MSI-high tumors. Given the low frequency of MSI-high colorectal tumors, results were matched with available mismatch repair deficiency data. Concordant with the smMIP-based NGS result, none of the 23 tested colorectal tumors showed mismatch repair deficiency by immunohistochemical staining of MLH1, PMS2, MSH2 and MSH6 (Supplementary Table 7). In addition, a supplementary set (*n* = 13) of mismatch repair deficient colorectal carcinomas as determined by IHC, was analyzed, confirming the assay sensitivity. All samples showed > 30% unstable loci and thus would have been classified as an MSI-high tumor by sequence analysis with the PATH panel (Supplementary Fig. 9). Taken together, identification of MSI in non-MSI-prone tumors shows the strength of combination analyses by one NGS panel. As MSI is the first tumor-agnostic FDA-approved genetic biomarker that gives access to therapy, identification of MSI is of importance for any cancer type [5, 51].

## Discussion

In the current study, a smMIP-based NGS approach was validated, which aimed at concurrent detection of genomic mutations, amplifications, and MSI in small amounts of histological tumor tissue or cytological material representing routinely available tumor samples. We provide detailed information on the validation of the procedure for predictive testing in current clinical practice and show that it is feasible to reliably analyze different types of genomic aberrations using a small smMIP-based NGS panel on diagnostic materials. Based on this sensitive and reliable molecular diagnostic analysis, cancer patients can be stratified for targeted therapies and immunotherapy regarding currently available drugs. The combined analysis of different types of genomic aberrations within one analysis saves valuable time, material, and costs. In addition, a broad predictive analysis allows identification of genomic aberrations that are rarely considered by clinicians, e.g. MSI in a non-MSI-prone tumor or a *KIT* mutation in melanoma, which might be beneficial for treatment options [5, 51, 52].

Previously, simultaneous detection of mutations with either amplifications or MSI using targeted NGS was described [10, 13, 19, 53–55]. To our knowledge this is the first small smMIP-based NGS panel that allows concurrent analysis of mutations, amplifications, and MSI by numerous microsatellite loci. Although whole-genome sequencing offers the most comprehensive analysis, the lack of fresh frozen material, high costs and low amounts of tissues/neoplastic cells hinder its use in routine diagnostics. Targeted NGS approaches on the other hand offer lower costs, can yield higher coverage for regions of interest, offer a fast turnaround time, and allow analysis of FFPE specimens with suboptimal gDNA quality. Our panel uses a PCR-based target enrichment method, which is suitable for small gene panels and can handle low (< 10 ng) gDNA input. Upscaling our routine diagnostic panel from the previously published Cancer Hotspot Panel (247 smMIPs [6]) to the PATH panel (663 smMIPs) did not affect sensitivity. In addition, the smMIP panel can be easily adapted by adding smMIPs that target new regions to an already optimized smMIP pool [6], which is an advance in the constantly changing field of personalized medicines and molecular markers, as was demonstrated by the addition of microsatellite markers. Nevertheless, the PATH panel does not prevent additional predictive analyses. Immunohistochemistry of PD-L1, ALK and ROS1, or mRNA based analysis to detect relevant fusion genes involving *ALK*, *ROS1*, *RET*, or *NTRK* genes cannot be replaced by the PATH panel [56]. In addition, tumor mutational burden, associated with a favorable response to immune checkpoint inhibitors [57, 58] cannot be performed by a small NGS panel. Although large (commercial) NGS panels (> 1 Mb) can be used for this analysis instead of whole-genome sequencing or whole-exome sequencing, these panels are generally more expensive, time consuming (due to the complexity of the analysis and interpretation of results), and not appropriate for all diagnostic requests.

The PATH panel was designed to cover most targetable mutations and amplifications for lung cancer, colorectal cancer, melanoma, and GIST for first line treatment options, as well in the setting of therapy resistance (e.g. *EGFR*, *ALK* and *KIT* gatekeeper mutations). In addition, the panel can be used to evaluate possible treatment options for other cancer types in a named or compassionate use program. The 729 diagnostic tumor samples that were evaluated with the smMIP panel showed 788 (likely) pathogenic mutations, including mutations that give access to targeted treatment options (e.g. mutations in *EGFR*, *BRAF*, *MET*, *ERBB2, KIT, PDGFRA*). Several large deletions and insertions in *MET* and *KIT* were found. Moreover, clinically relevant amplifications were identified, including targetable amplifications (i.e. *MET*, *ERBB2*, *FGFR1*) and amplifications (*MDM2*) associated with hyperprogressive disease upon immunotherapy treatment [33, 44–46, 48, 50, 59]. Targetable aberrations were not only detected in lung cancer, colorectal cancer, melanoma and GIST, but also in the other cancer types. Lastly, MSI was observed in both MSI-prone and non-MSI-prone tumor samples, which predicts response to immunotherapy [5, 51]. These results show that with the PATH panel a broad spectrum of actionable genetic alterations can be evaluated.

During validation, cut-offs for gene amplifications and MSI analysis were established. In addition, we have identified pitfalls of these analyses. Amplification analysis by NGS relies on sequencing coverage possibly combined with VAFs of germline polymorphisms (SNPs) [11]. We based our analysis on sequencing coverage. Before coverage outliers can be detected, a suitable normalization method has to be applied. The median coverage was shown to be a convenient normalization method, whereas the mean and summed coverage appeared less suitable. To detect coverage outliers, coverage in the sample can be compared to normal samples in the same sequencing run or an external reference pool [10, 60–62]. This latter one saves valuable costs and time (due to lack of availability of a normal sample for every tumor sample) and was shown to generate reliable results. Detection of outliers can be achieved by determining a threshold [54, 63, 64], calculating a *p*-value [7, 61], or bootstrap based estimation of the confidence intervals [65]. We chose for the threshold method and validated a relative coverage ≥3.0. This method allows quantification of amplifications by deducing the number of alleles in the tumor cells from the relative coverage and tumor purity of the sample [11]. Accordingly the clinical relevance of amplifications can be established (i.e. low or high number amplification). In addition, the number of gene copies that needs to be present to be quantifiable by the NGS analysis can be deduced and should be considered to be included in the diagnostic report to specify restrictions of NGS-based detection of copy number gains [11]. Although these deductions depend on the estimated tumor purity, which is error-prone, the VAF of somatic variants can be used to support this estimation. If a low sensitivity is obtained or low-level amplifications are of interest, one could consider FISH analysis to detect a specific amplification or OncoScan array to comprehensively analyze copy number variations. Despite that these analyses are more sensitive to exclude the presence of amplifications, especially in samples with limited tumor load and to detect low level amplifications or amplifications in a subset of tumor cells with clusters, the smMIP-based NGS based analysis offers a cost-effective multiplex approach.

Like amplification analysis, MSI analysis was established without the need for a paired normal sample. The five markers that are routinely studied by the pentaplex PCR are mainly validated in Lynch syndrome associated tumors. This approach is not necessarily applicable to NGS based analysis on different tumor types. With respect to the tumor type, different microsatellites might be affected. Indeed, studies focusing on the spectrum of unstable microsatellites across multiple cancer types showed different patterns of MSI [15–17]. The NGS based methodology also differs from the fragment length analysis based read out. In the smMIP-based NGS panel 55 microsatellite repeats were included that were reported unstable across multiple cancer types in NGS based analysis [15, 16]. By increasing the number of microsatellites that are evaluated, the smMIP-based NGS panel may be more sensitive for a broad range of tumor types compared to the traditional pentaplex PCR. If new predictive microsatellite loci are identified in future studies, smMIPs that target these microsatellite regions can be added to the smMIP pool. Tumor purity also contributes to the sensitivity of the assay. We determined a cut-off of at least 30% tumor cells to reliably exclude MSI and a fraction of unstable loci of over 30% to classify a sample as MSI. The relative low number of MSI-high samples in colorectal carcinoma urged additional validation which confirmed the assay sensitivity. The low frequency of MSI in the colorectal carcinoma samples in the diagnostic cohort, is in line with a low frequency of MSI-high tumors among metastasized colorectal cancer cases [66].

## Conclusion

More genomic information can be extracted from tumor samples than ever before, but less material is available for testing. In the current study, we show that reliable, sensitive, and simultaneous detection of mutations, gene amplifications, and MSI can be achieved in routinely available diagnostic tumor samples using an integrated smMIP-based NGS approach, which was designed in close collaboration with clinicians and molecular biologists of several specialized centers. This single smMIP-based NGS test reduces the number of analyses that are typically performed on tumor samples, uses only limited amount of material, and thereby simplifies the workflow for molecular cancer diagnostics. Moreover, the panel allows easy adaptation and can thereby comply with the rapidly expanding molecular markers.

## Supplementary information

**Additional file 1:.** This files contains supplementary Fig. 1–9 and the supplementary figure legends.

**Additional file 2:.** This files contains supplementary Table 1–8.

**Additional file 3:.** This files contains the references of supplementary Table 1.

## Data Availability

The datasets used and/or analysed during the current study are available from the corresponding author on reasonable request.

## Abbreviations

CNVs: Copy-number variations
FISH: Fluorescence in situ hybridization
GIST: Gastro-intestinal stromal tumors
IHC: Immunohistochemistry
MSI: Microsatellite instability
MSS: Microsatellite stable
NGS: Next-generation sequencing
PATH: Predictive Analysis for Therapy
smMIP: Single-molecule molecular inversion probe
VAF: Variant allele frequency
VUS: Variant of unknown significance
