# Supplementary material for: Comprehensive routine diagnostic screening to identify predictive mutations, gene amplifications, and microsatellite instability in FFPE tumor material
Source: BMC Cancer. 2020 Apr 7;20:291. doi: 10.1186/s12885-020-06785-6 (PMC7137451; doi:10.1186/s12885-020-06785-6)
Supplement: Supplementary file 1 — Additional file 1:. This files contains supplementary Fig. 1–9 and the supplementary figure legends. [file 12885_2020_6785_MOESM1_ESM.pdf]

Supplementary Figure 1-9

Comprehensive routine diagnostic screening to identify predictive mutations, gene amplifications, and microsatellite instability in FFPE tumor material

Elisabeth M.P. Steeghs[1,*], Leonie I. Kroeze[1,*], Bastiaan B.J. Tops[1,2], Leon C. van Kempen[3], Arja ter Elst[3], Annemiek W.M. Kastner-van Raaij[1], Sandra J.B. Hendriks-Cornelissen[1], Mandy J.W. Hermsen[1], Erik A.M. Jansen[5], Petra M. Nederlof [4], Ed Schuuring[3], Marjolijn J.L. Ligtenberg[1,5,*], Astrid Eijkelenboom[1,*]

** authors contributed equally*

[1] Department of Pathology, Radboud university medical center, Nijmegen, The Netherlands

[2] Department of Pathology, Princess Máxima Center for Pediatric Oncology, Utrecht, The Netherlands

[3] Department of Pathology, University Medical Center Groningen, University of Groningen, Groningen, The Netherlands

[4] Department of Pathology, The Netherlands Cancer Institute, Amsterdam, The Netherlands

[5] Department of Human Genetics, Radboud university medical center, Nijmegen, The Netherlands

Corresponding author:
Astrid Eijkelenboom, PhD
Radboud University Medical Center
PO Box 9101, 6500 HB, Nijmegen, the Netherlands
E-mail: Astrid.Eijkelenboom@radboudumc.nl
Phone: 0031-243655374, Fax: 0031-243668750

Supplementary Figure 1

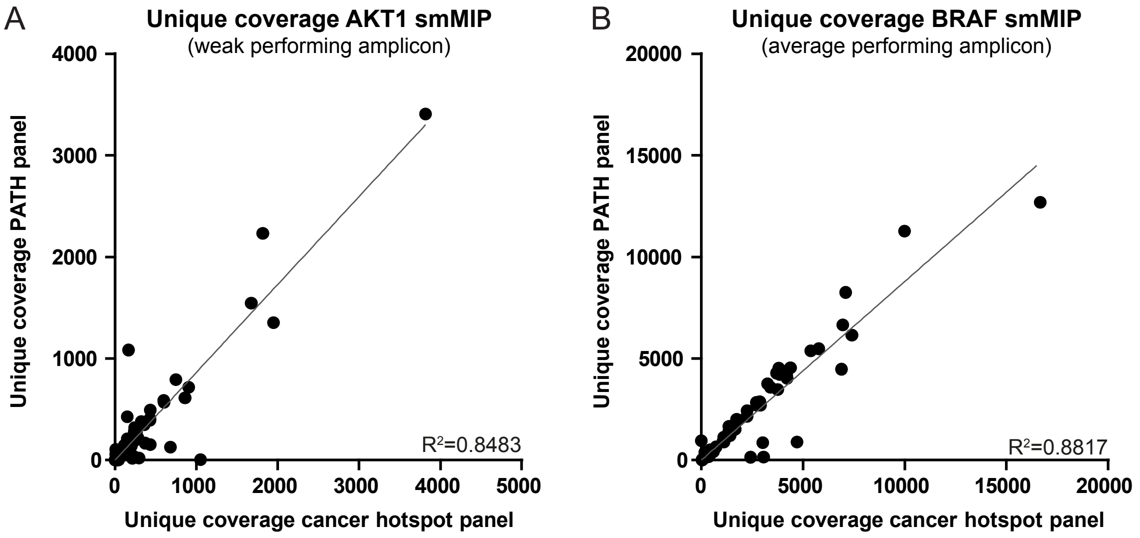

Supplementary Figure 2

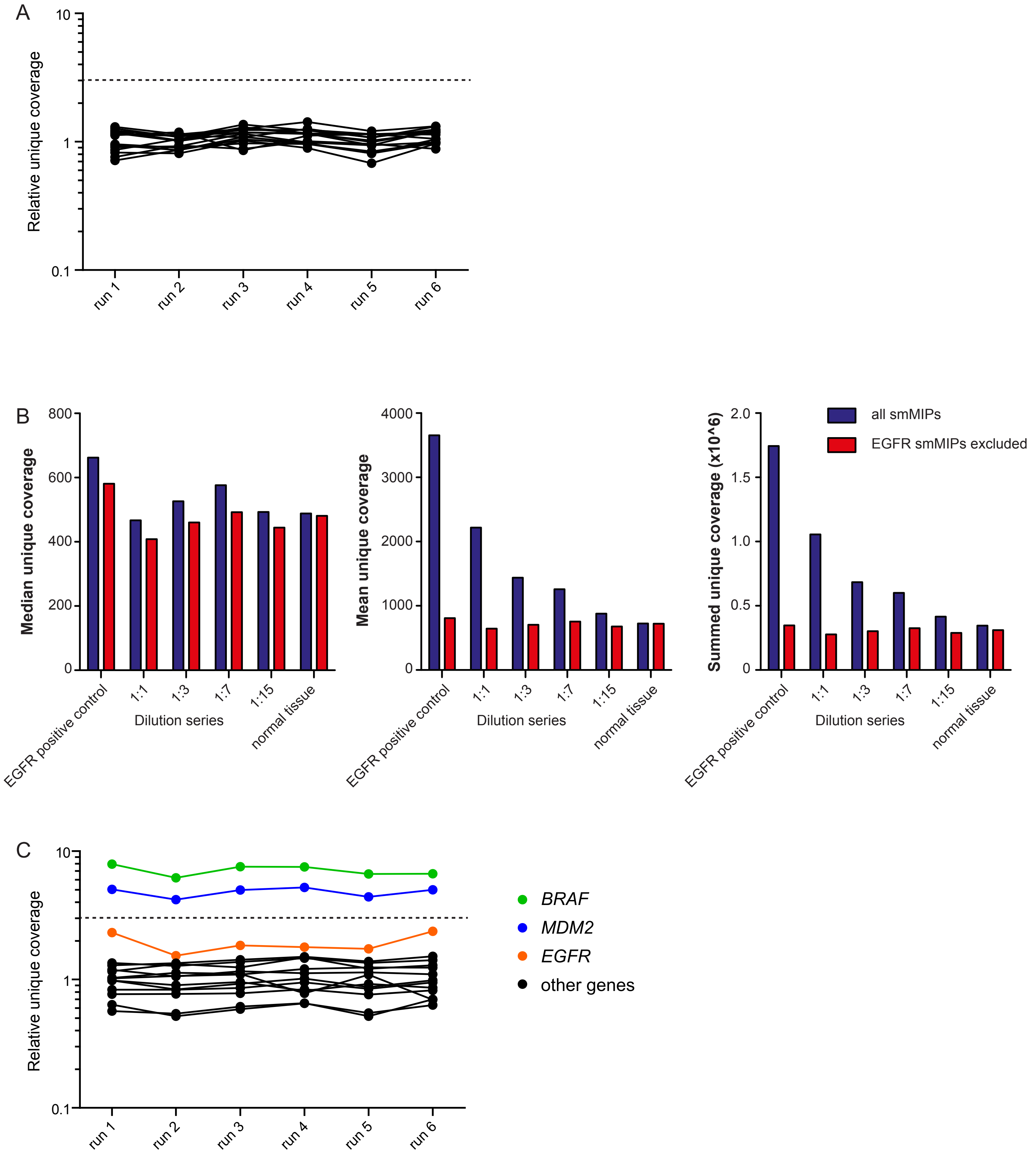

Supplementary Figure 3

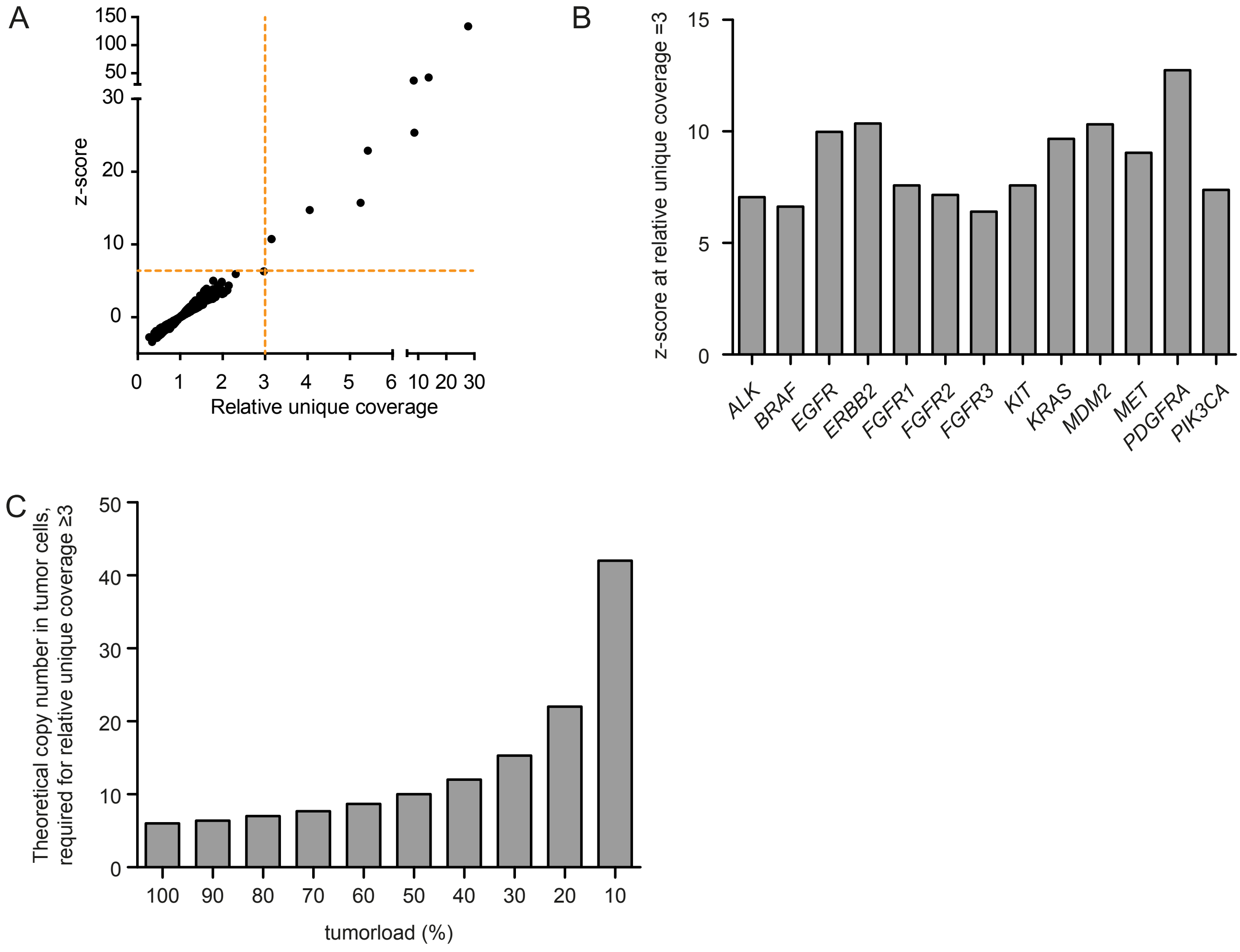

Supplementary Figure 4

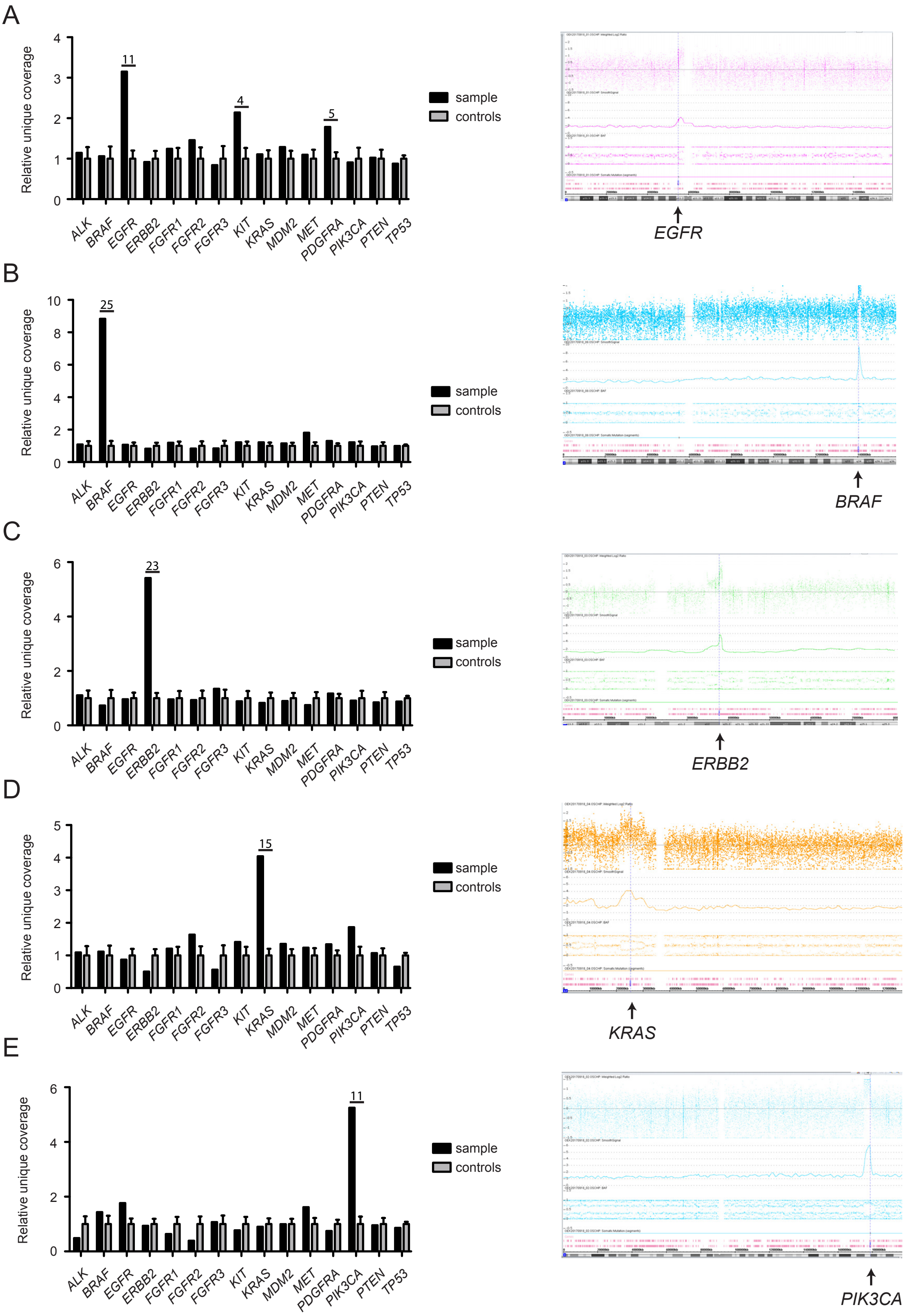

Supplementary Figure 5

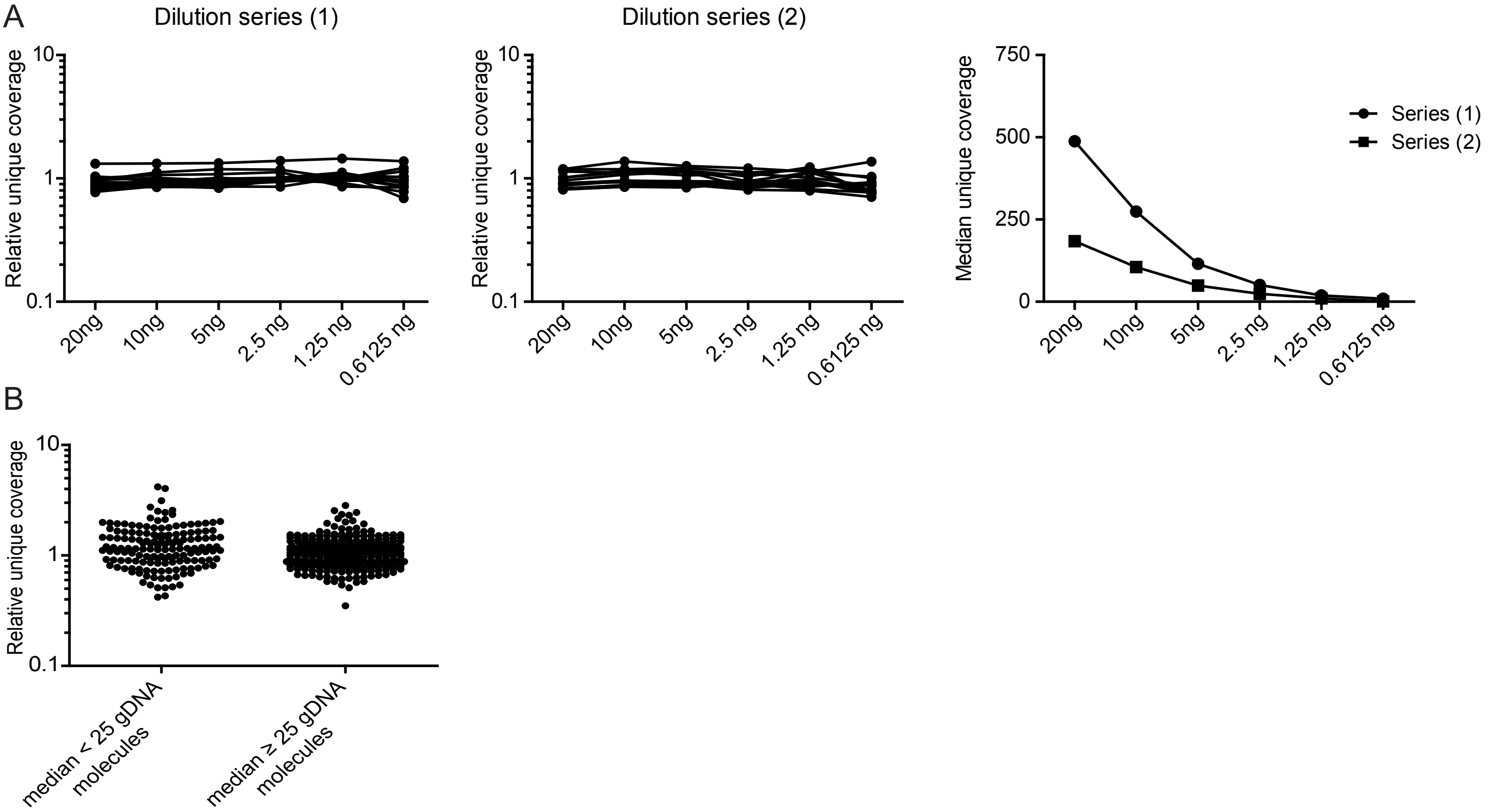

Supplementary Figure 6

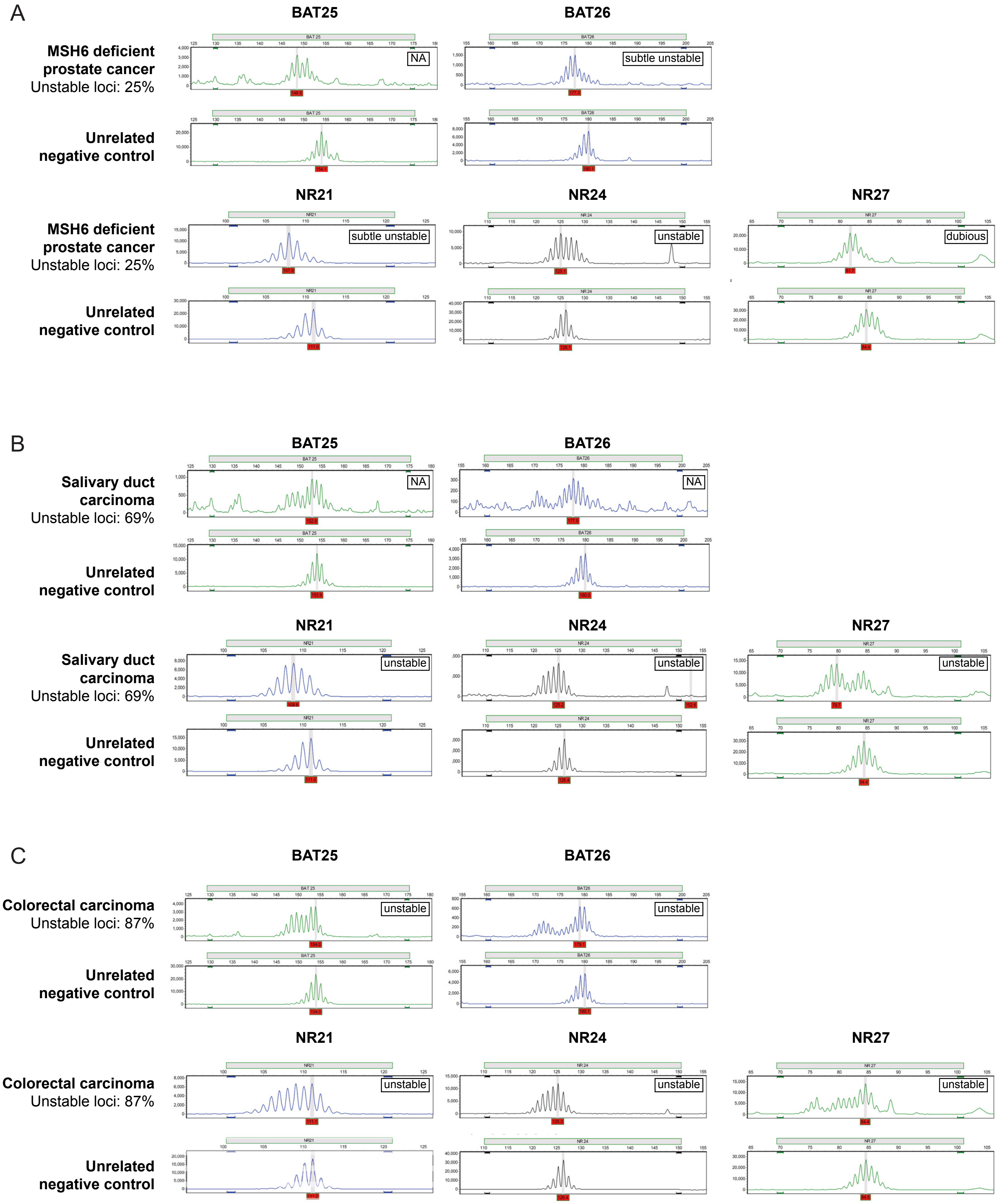

Supplementary Figure 7

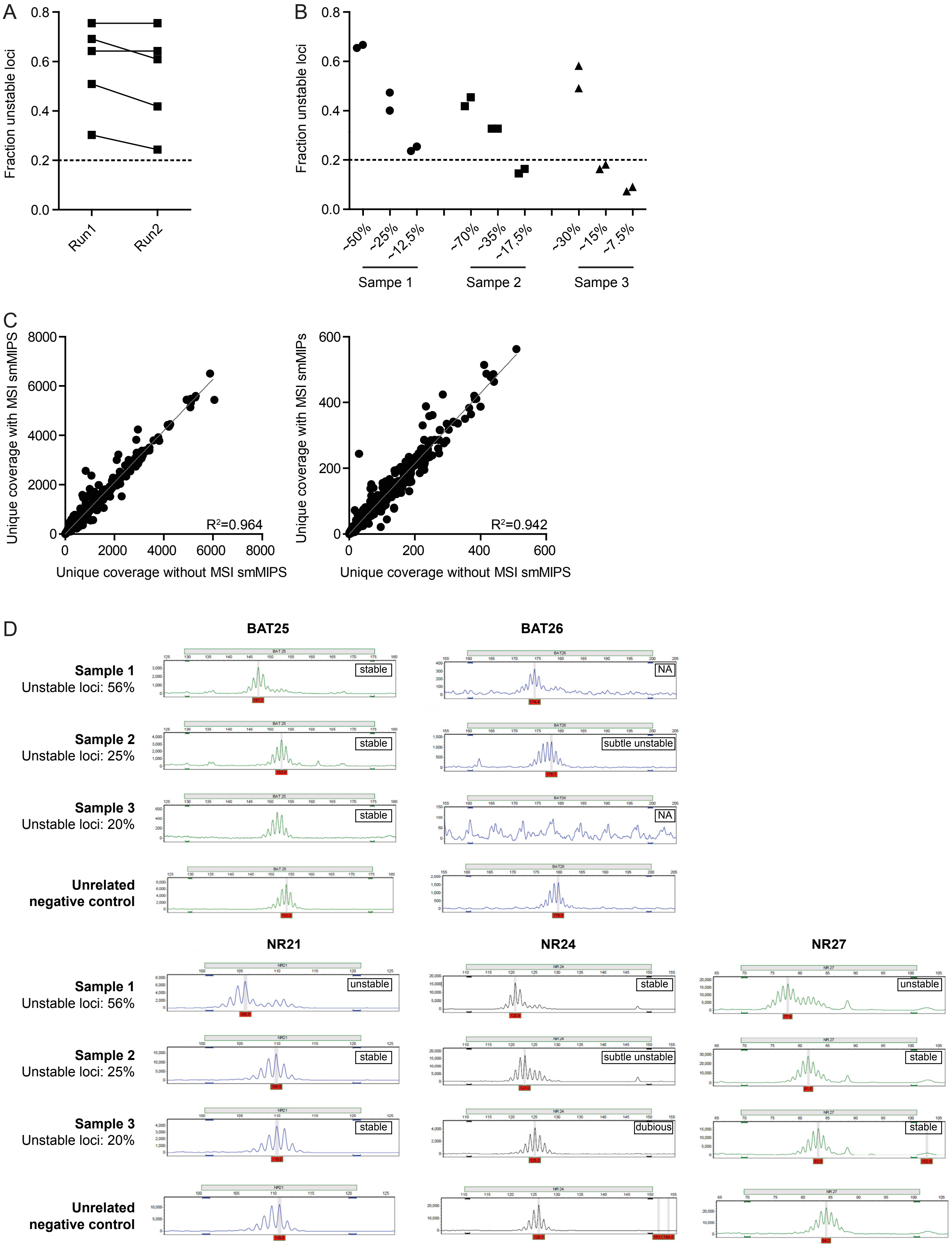

Supplementary Figure 8

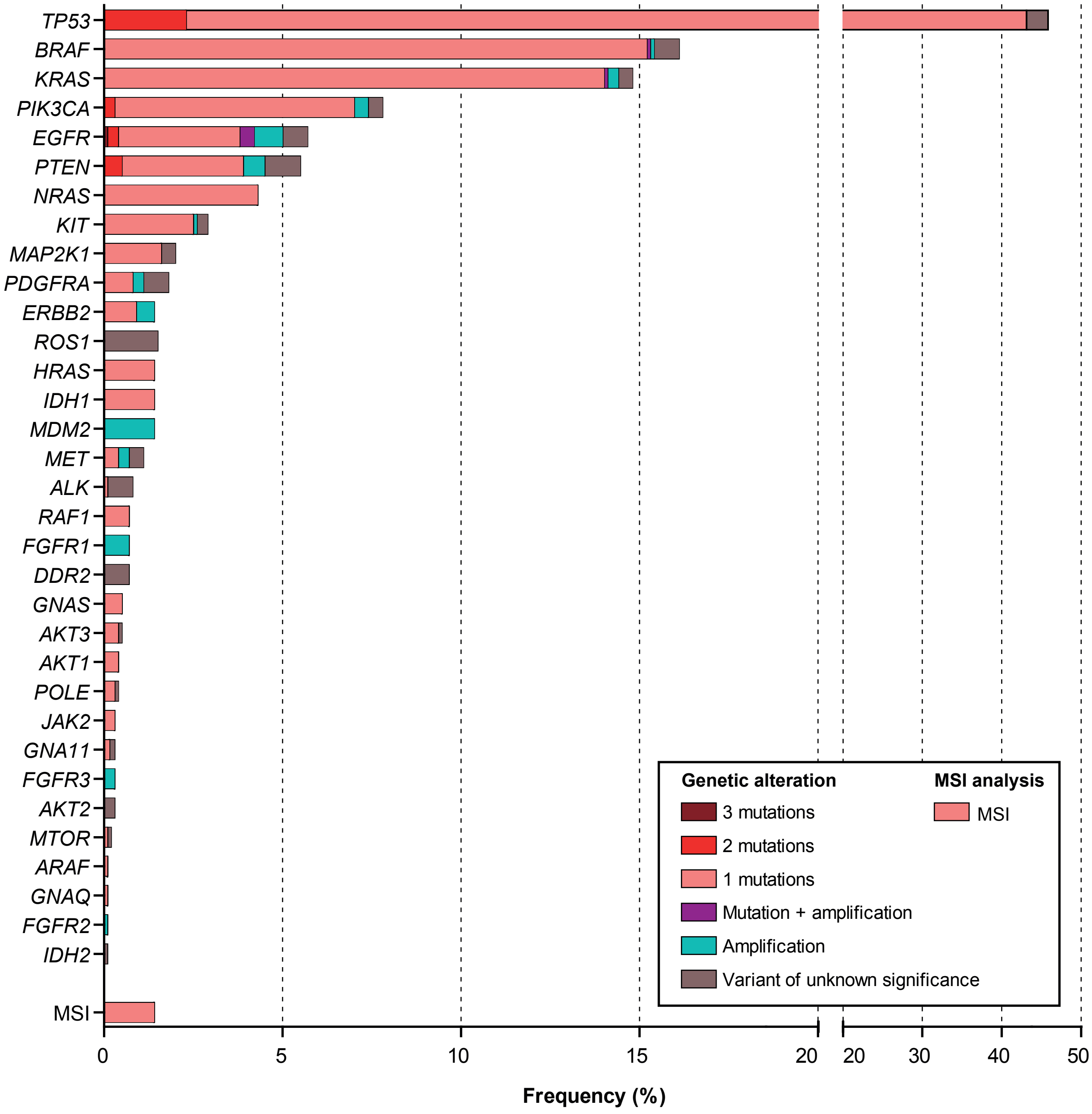

Supplementary Figure 9

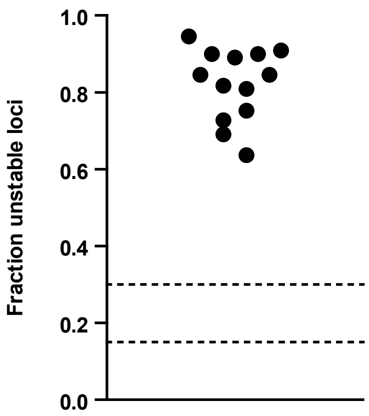

Supplementary Figure Legends

Supplementary Figure 1. Association in coverage between the PATH and cancer hotspot panel.

Parallel analyses of the cancer hotspot panel and PATH panel (both single analysis) showed a comparable unique coverage for the individual regions. Comparison of the coverage of (**A**) a weakly performing AKT1 smMIP and (**B**) an average performing BRAF smMIP is shown.

Supplementary Figure 2. Stability of the CNV analysis.

(**A**) Relative unique coverage of a negative normal FFPE tissue gDNA control, which was analyzed in separate diagnostic library preparations and NGS runs. For each run, the coverage relative to the independent control group is shown for all genes. (**B**) The unique coverage of all smMIPs in analysis of gDNA from the *EGFR* amplified positive control and a dilution series into gDNA obtained from normal tissue is determined. The median, mean, or summed unique coverage is calculated over all smMIPs in the panel or all smMIPs excluding the EGFR targeting smMIPs and represented in the graphs. (**C**) Relative unique coverage of a mixed positive control FFPE gDNA sample, containing a *BRAF, MDM2* and low level *EGFR* amplification. The sample was analyzed in separate diagnostic library preparations and NGS runs. For each run, the coverage relative to the independent control group is shown for all genes. The dashed line indicates the validated threshold (relative coverage ≥3) for detection of amplifications.

Supplementary Figure 3. Consequences of threshold ≥3 in relative coverage.

(**A**) Scatter plot of relative unique coverage and z-score in 46 clinical samples. The cut-offs for validation (relative coverage ≥ 3.0 and z-score > 6.4) are shown by an orange lines. (**B**) Accompanying z-scores per gene at a relative coverage of 3.0. (**C**) Required number of copies in the tumor cell allowing detection of amplification with a relative coverage ≥3.0.

Supplementary Figure 4. OncoScan-array analysis confirms detected amplifications.

On the left, the relative unique coverages per gene are shown per diagnostic sample compared to the control series. In addition, the z-score of the amplified genes are shown above the bars. On the right the genomic location and surrounding sequences for the potential amplified gene in OncoScan-array analysis is shown. (**A**)

EGFR positive control. (**B**) *BRAF* positive control. (**C**) *ERBB2* positive control. (**D**) *KRAS* positive control. (**E**) *PIK3CA* positive control.

Supplementary Figure 5. CNV calling in low coverage analyses.

(**A**) Two normal tissue negative control samples were diluted (concentrations indicated below axis) and the relative unique coverage of all 15 genes is shown for both individual series. The median unique coverage decreases with decreased input (figure to the right). (**B**) All validation series analyses were grouped based on median unique coverage <25 and ≥25 gDNA molecules analyzed per amplicon. Samples with evident amplifications were removed from the analyses.

Supplementary Figure 6. MSI positive controls for MSI analysis by the PATH panel.

(**A-C**) PCR pentaplex results of three positive control samples (prostate cancer, salivary duct carcinoma, and colorectal carcinoma) for the validation of the MSI analysis by the PATH panel. The percentage of unstable microsatellites obtained from the smMIP-based NGS analysis (25% (**A**), 69% (**B**), and 87%) are depicted.

Supplementary Figure 7. Validation of MSI analyses by the PATH panel.

(**A**) 5 positive control samples were analyzed in two independent runs. Fraction of unstable loci of both analyses are shown. (**B**) Three positive control samples were diluted in gDNA isolated from normal tissue. The fraction of unstable loci of duplicate analyses are depicted. On the y-axis the estimated percentage of tumor cells is depicted. (**C**) Parallel analyses of two samples by PATH panel with or without smMIPs for MSI detection. Both samples showed a comparable coverage for the individual regions. (**D**) Validation of MSI analysis by pentaplex PCR. MSI status was validated by PCR pentaplex analyses of three samples showed ≥ 20% unstable loci.

Supplementary Figure 8. Frequency of genetic alterations detected by the PATH panel.

Mutations, amplification, and MSI status of 729 diagnostic tumor samples, which were sequenced with the PATH panel. Genes are sorted based on the occurrence of mutations and amplifications.

Supplementary Figure 9. Validation of MSI analyses in colorectal carcinoma samples.

The fraction of microsatellite loci that showed an MSI event is depicted for 14 MSI colorectal samples (IHC: loss of MLH1 and PMS2).
