# Supplementary material for: Comprehensive routine diagnostic screening to identify predictive mutations, gene amplifications, and microsatellite instability in FFPE tumor material
Source: BMC Cancer. 2020 Apr 7;20:291. doi: 10.1186/s12885-020-06785-6 (PMC7137451; doi:10.1186/s12885-020-06785-6)
Supplement: Supplementary file 3 — Additional file 3:. This files contains the references of supplementary Table 1. [file 12885_2020_6785_MOESM3_ESM.pdf]

Supplementary data: References Supplementary Table 1

Comprehensive routine diagnostic screening to identify predictive mutations, gene amplifications, and microsatellite instability in FFPE tumor material

Elisabeth M.P. Steeghs[1,*], Leonie I. Kroeze[1,*], Bastiaan B.J. Tops[1,2], Leon C. van Kempen[3], Arja ter Elst[3], Annemiek W.M. Kastner-van Raaij[1], Sandra J.B. Hendriks-Cornelissen[1], Mandy J.W. Hermsen[1], Erik A.M. Jansen[5], Petra M. Nederlof [4], Ed Schuuring[3], Marjolijn J.L. Ligtenberg[1,5,*], Astrid Eijkelenboom[1,*]

** authors contributed equally*

[1] Department of Pathology, Radboud university medical center, Nijmegen, The Netherlands

[2] Department of Pathology, Princess Máxima Center for Pediatric Oncology, Utrecht, The Netherlands

[3] Department of Pathology, University Medical Center Groningen, University of Groningen, Groningen, The Netherlands

[4] Department of Pathology, The Netherlands Cancer Institute, Amsterdam, The Netherlands

[5] Department of Human Genetics, Radboud university medical center, Nijmegen, The Netherlands

Corresponding author:
Astrid Eijkelenboom, PhD
Radboud University Medical Center
PO Box 9101, 6500 HB, Nijmegen, the Netherlands
E-mail: Astrid.Eijkelenboom@radboudumc.nl
Phone: 0031-243655374, Fax: 0031-243668750

1. Carpten JD, Faber AL, Horn C, Donoho GP, Briggs SL, Robbins CM, Hostetter G, Boguslawski S, Moses TY, Savage S *et al*: **A transforming mutation in the pleckstrin homology domain of AKT1 in cancer**. *Nature* 2007, **448**(7152):439-444.

2. Yi KH, Lauring J: **Recurrent AKT mutations in human cancers: functional consequences and effects on drug sensitivity**. *Oncotarget* 2016, **7**(4):4241-4251.

3. Parikh C, Janakiraman V, Wu WI, Foo CK, Kljavin NM, Chaudhuri S, Stawiski E, Lee B, Lin J, Li H *et al*: **Disruption of PH-kinase domain interactions leads to oncogenic activation of AKT in human cancers**. *Proc Natl Acad Sci U S A* 2012, **109**(47):19368-19373.

4. Davies MA, Stemke-Hale K, Tellez C, Calderone TL, Deng W, Prieto VG, Lazar AJ, Gershenwald JE, Mills GB: **A novel AKT3 mutation in melanoma tumours and cell lines**. *Br J Cancer* 2008, **99**(8):1265-1268.

5. Holla VR, Elamin YY, Bailey AM, Johnson AM, Litzenburger BC, Khotskaya YB, Sanchez NS, Zeng J, Shufean MA, Shaw KR *et al*: **ALK: a tyrosine kinase target for cancer therapy**. *Cold Spring Harb Mol Case Stud* 2017, **3**(1):a001115.

6. Drizou M, Kotteas EA, Syrigos N: **Treating patients with ALK-rearranged non-small-cell lung cancer: mechanisms of resistance and strategies to overcome it**. *Clin Transl Oncol* 2017, **19**(6):658-666.

7. Salido M, Pijuan L, Martinez-Aviles L, Galvan AB, Canadas I, Rovira A, Zanui M, Martinez A, Longaron R, Sole F *et al*: **Increased ALK gene copy number and amplification are frequent in non-small cell lung cancer**. *J Thorac Oncol* 2011, **6**(1):21-27.

8. Katayama R: **Therapeutic strategies and mechanisms of drug resistance in anaplastic lymphoma kinase (ALK)-rearranged lung cancer**. *Pharmacol Ther* 2017, **177**:1-8.

9. Imielinski M, Greulich H, Kaplan B, Araujo L, Amann J, Horn L, Schiller J, Villalona-Calero MA, Meyerson M, Carbone DP: **Oncogenic and sorafenib-sensitive ARAF mutations in lung adenocarcinoma**. *J Clin Invest* 2014, **124**(4):1582-1586.

10. Shi H, Moriceau G, Kong X, Lee MK, Lee H, Koya RC, Ng C, Chodon T, Scolyer RA, Dahlman KB *et al*: **Melanoma whole-exome sequencing identifies (V600E)B-RAF amplification-mediated acquired B-RAF inhibitor resistance**. *Nat Commun* 2012, **3**:724.

11. Tsao H, Chin L, Garraway LA, Fisher DE: **Melanoma: from mutations to medicine**. *Genes Dev* 2012, **26**(11):1131-1155.

12. Sasaki H, Maekawa M, Tatematsu T, Okuda K, Moriyama S, Yano M, Fujii Y: **Increased BRAF copy number in lung adenocarcinoma**. *Oncol Lett* 2015, **9**(2):709-712.

13. Wilson MA, Zhao F, Khare S, Roszik J, Woodman SE, D'Andrea K, Wubbenhorst B, Rimm DL, Kirkwood JM, Kluger HM *et al*: **Copy Number Changes Are Associated with Response to Treatment with Carboplatin, Paclitaxel, and Sorafenib in Melanoma**. *Clin Cancer Res* 2016, **22**(2):374-382.

14. Barras D: **BRAF Mutation in Colorectal Cancer: An Update**. *Biomark Cancer* 2015, **7**(Suppl 1):9-12.

15. Beauchamp EM, Woods BA, Dulak AM, Tan L, Xu C, Gray NS, Bass AJ, Wong KK, Meyerson M, Hammerman PS: **Acquired resistance to dasatinib in lung cancer cell lines conferred by DDR2 gatekeeper mutation and NF1 loss**. *Mol Cancer Ther* 2014, **13**(2):475-482.

16. Hammerman PS, Sos ML, Ramos AH, Xu C, Dutt A, Zhou W, Brace LE, Woods BA, Lin W, Zhang J *et al*: **Mutations in the DDR2 kinase gene identify a novel therapeutic target in squamous cell lung cancer**. *Cancer Discov* 2011, **1**(1):78-89.

17. Lee MS, Jung EA, An SB, Kim YJ, Oh DY, Song JY, Um SW, Han J, Choi YL: **Prevalence of Mutations in Discoidin Domain-Containing Receptor Tyrosine Kinase 2 (DDR2) in Squamous Cell Lung Cancers in Korean Patients**. *Cancer Res Treat* 2017, **49**(4):1065-1076.

18. Miao L, Wang Y, Zhu S, Shi M, Li Y, Ding J, Yang J, Ye Q, Cai H, Zhang D *et al*: **Identification of novel driver mutations of the discoidin domain receptor 2 (DDR2) gene in squamous cell lung cancer of Chinese patients**. *BMC Cancer* 2014, **14**:369.

19. Bertotti A, Papp E, Jones S, Adleff V, Anagnostou V, Lupo B, Sausen M, Phallen J, Hruban CA, Tokheim C *et al*: **The genomic landscape of response to EGFR blockade in colorectal cancer**. *Nature* 2015, **526**(7572):263-267.

20. Gan HK, Cvrljevic AN, Johns TG: **The epidermal growth factor receptor variant III (EGFRvIII): where wild things are altered**. *FEBS J* 2013, **280**(21):5350-5370.

21. Metzger B, Chambeau L, Begon DY, Faber C, Kayser J, Berchem G, Pauly M, Boniver J, Delvenne P, Dicato M *et al*: **The human epidermal growth factor receptor (EGFR) gene in European patients with advanced colorectal cancer harbors infrequent mutations in its tyrosine kinase domain**. *BMC Med Genet* 2011, **12**:144.

22. Peciak J, Stec WJ, Treda C, Ksiazkiewicz M, Janik K, Popeda M, Smolarz M, Rosiak K, Hulas-Bigoszewska K, Och W *et al*: **Low Incidence along with Low mRNA Levels of EGFRvIII in Prostate and Colorectal Cancers Compared to Glioblastoma**. *J Cancer* 2017, **8**(1):146-151.

23. Sasaki H, Kawano O, Endo K, Yukiue H, Yano M, Fujii Y: **EGFRvIII mutation in lung cancer correlates with increased EGFR copy number**. *Oncol Rep* 2007, **17**(2):319-323.

24. Kavuri SM, Jain N, Galimi F, Cottino F, Leto SM, Migliardi G, Searleman AC, Shen W, Monsey J, Trusolino L *et al*: **HER2 activating mutations are targets for colorectal cancer treatment**. *Cancer Discov* 2015, **5**(8):832-841.

25. Chmielecki J, Ross JS, Wang K, Frampton GM, Palmer GA, Ali SM, Palma N, Morosini D, Miller VA, Yelensky R *et al*: **Oncogenic alterations in ERBB2/HER2 represent potential therapeutic targets across tumors from diverse anatomic sites of origin**. *Oncologist* 2015, **20**(1):7-12.

26. Herter-Sprie GS, Greulich H, Wong KK: **Activating Mutations in ERBB2 and Their Impact on Diagnostics and Treatment**. *Front Oncol* 2013, **3**:86.

27. Seo AN, Kwak Y, Kim DW, Kang SB, Choe G, Kim WH, Lee HS: **HER2 status in colorectal cancer: its clinical significance and the relationship between HER2 gene amplification and expression**. *PLoS One* 2014, **9**(5):e98528.

28. Takezawa K, Pirazzoli V, Arcila ME, Nebhan CA, Song X, de Stanchina E, Ohashi K, Janjigian YY, Spitzler PJ, Melnick MA *et al*: **HER2 amplification: a potential mechanism of acquired resistance to EGFR inhibition in EGFR-mutant lung cancers that lack the second-site EGFRT790M mutation**. *Cancer Discov* 2012, **2**(10):922-933.

29. Helsten T, Elkin S, Arthur E, Tomson BN, Carter J, Kurzrock R: **The FGFR Landscape in Cancer: Analysis of 4,853 Tumors by Next-Generation Sequencing**. *Clin Cancer Res* 2016, **22**(1):259-267.

30. Hibi M, Kaneda H, Tanizaki J, Sakai K, Togashi Y, Terashima M, De Velasco MA, Fujita Y, Banno E, Nakamura Y *et al*: **FGFR gene alterations in lung squamous cell carcinoma are potential targets for the multikinase inhibitor nintedanib**. *Cancer Sci* 2016, **107**(11):1667-1676.

31. Katoh M, Nakagama H: **FGF receptors: cancer biology and therapeutics**. *Med Res Rev* 2014, **34**(2):280-300.

32. Pfarr N, Penzel R, Klauschen F, Heim D, Brandt R, Kazdal D, Jesinghaus M, Herpel E, Schirmacher P, Warth A *et al*: **Copy number changes of clinically actionable genes in melanoma, non-small cell lung

cancer and colorectal cancer-A survey across 822 routine diagnostic cases. *Genes Chromosomes Cancer* 2016, **55**(11):821-833.

33. O'Hayre M, Vazquez-Prado J, Kufareva I, Stawiski EW, Handel TM, Seshagiri S, Gutkind JS: **The emerging mutational landscape of G proteins and G-protein-coupled receptors in cancer**. *Nat Rev Cancer* 2013, **13**(6):412-424.

34. van Dijk MC, Bernsen MR, Ruiter DJ: **Analysis of mutations in B-RAF, N-RAS, and H-RAS genes in the differential diagnosis of Spitz nevus and spitzoid melanoma**. *Am J Surg Pathol* 2005, **29**(9):1145-1151.

35. van Engen-van Grunsven AC, van Dijk MC, Ruiter DJ, Klaasen A, Mooi WJ, Blokx WA: **HRAS-mutated Spitz tumors: A subtype of Spitz tumors with distinct features**. *Am J Surg Pathol* 2010, **34**(10):1436-1441.

36. Shibata T, Kokubu A, Miyamoto M, Sasajima Y, Yamazaki N: **Mutant IDH1 confers an in vivo growth in a melanoma cell line with BRAF mutation**. *Am J Pathol* 2011, **178**(3):1395-1402.

37. Lopez GY, Reitman ZJ, Solomon D, Waldman T, Bigner DD, McLendon RE, Rosenberg SA, Samuels Y, Yan H: **IDH1(R132) mutation identified in one human melanoma metastasis, but not correlated with metastases to the brain**. *Biochem Biophys Res Commun* 2010, **398**(3):585-587.

38. Lipson D, Capelletti M, Yelensky R, Otto G, Parker A, Jarosz M, Curran JA, Balasubramanian S, Bloom T, Brennan KW *et al*: **Identification of new ALK and RET gene fusions from colorectal and lung cancer biopsies**. *Nat Med* 2012, **18**(3):382-384.

39. Dumaz N, Andre J, Sadoux A, Laugier F, Podgorniak MP, Mourah S, Lebbe C: **Driver KIT mutations in melanoma cluster in four hotspots**. *Melanoma Res* 2015, **25**(1):88-90.

40. Todd JR, Becker TM, Kefford RF, Rizos H: **Secondary c-Kit mutations confer acquired resistance to RTK inhibitors in c-Kit mutant melanoma cells**. *Pigment Cell Melanoma Res* 2013, **26**(4):518-526.

41. Pierotti MA, Tamborini E, Negri T, Pricl S, Pilotti S: **Targeted therapy in GIST: in silico modeling for prediction of resistance**. *Nat Rev Clin Oncol* 2011, **8**(3):161-170.

42. Beadling C, Jacobson-Dunlop E, Hodi FS, Le C, Warrick A, Patterson J, Town A, Harlow A, Cruz F, 3rd, Azar S *et al*: **KIT gene mutations and copy number in melanoma subtypes**. *Clin Cancer Res* 2008, **14**(21):6821-6828.

43. Karachaliou N, Mayo C, Costa C, Magri I, Gimenez-Capitan A, Molina-Vila MA, Rosell R: **KRAS mutations in lung cancer**. *Clin Lung Cancer* 2013, **14**(3):205-214.

44. Misale S, Yaeger R, Hobor S, Scala E, Janakiraman M, Liska D, Valtorta E, Schiavo R, Buscarino M, Siravegna G *et al*: **Emergence of KRAS mutations and acquired resistance to anti-EGFR therapy in colorectal cancer**. *Nature* 2012, **486**(7404):532-536.

45. Wagner PL, Stiedl AC, Wilbertz T, Petersen K, Scheble V, Menon R, Reischl M, Mikut R, Rubin MA, Fend F *et al*: **Frequency and clinicopathologic correlates of KRAS amplification in non-small cell lung carcinoma**. *Lung Cancer* 2011, **74**(1):118-123.

46. Valtorta E, Misale S, Sartore-Bianchi A, Nagtegaal ID, Paraf F, Lauricella C, Dimartino V, Hobor S, Jacobs B, Ercolani C *et al*: **KRAS gene amplification in colorectal cancer and impact on response to EGFR-targeted therapy**. *Int J Cancer* 2013, **133**(5):1259-1265.

47. Arcila ME, Drilon A, Sylvester BE, Lovly CM, Borsu L, Reva B, Kris MG, Solit DB, Ladanyi M: **MAP2K1 (MEK1) Mutations Define a Distinct Subset of Lung Adenocarcinoma Associated with Smoking**. *Clin Cancer Res* 2015, **21**(8):1935-1943.

48. Marks JL, Gong Y, Chitale D, Golas B, McLellan MD, Kasai Y, Ding L, Mardis ER, Wilson RK, Solit D *et al*: **Novel MEK1 mutation identified by mutational analysis of epidermal growth factor receptor signaling pathway genes in lung adenocarcinoma**. *Cancer Res* 2008, **68**(14):5524-5528.

49. Nikolaev SI, Rimoldi D, Iseli C, Valsesia A, Robyr D, Gehrig C, Harshman K, Guipponi M, Bukach O, Zoete V *et al*: **Exome sequencing identifies recurrent somatic MAP2K1 and MAP2K2 mutations in melanoma**. *Nat Genet* 2011, **44**(2):133-139.

50. Dworakowska D, Jassem E, Jassem J, Peters B, Dziadziuszko R, Zylicz M, Jakobkiewicz-Banecka J, Kobierska-Gulida G, Szymanowska A, Skokowski J *et al*: **MDM2 gene amplification: a new independent factor of adverse prognosis in non-small cell lung cancer (NSCLC)**. *Lung Cancer* 2004, **43**(3):285-295.

51. Hav M, Libbrecht L, Ferdinande L, Pattyn P, Laurent S, Peeters M, Praet M, Pauwels P: **MDM2 gene amplification and protein expressions in colon carcinoma: is targeting MDM2 a new therapeutic option?** *Virchows Arch* 2011, **458**(2):197-203.

52. Tornillo L, Duchini G, Carafa V, Lugli A, Dirnhofer S, Di Vizio D, Boscaino A, Russo R, Tapia C, Schneider-Stock R *et al*: **Patterns of gene amplification in gastrointestinal stromal tumors (GIST)**. *Lab Invest* 2005, **85**(7):921-931.

53. Awad MM, Oxnard GR, Jackman DM, Savukoski DO, Hall D, Shivdasani P, Heng JC, Dahlberg SE, Janne PA, Verma S *et al*: **MET Exon 14 Mutations in Non-Small-Cell Lung Cancer Are Associated With Advanced Age and Stage-Dependent MET Genomic Amplification and c-Met Overexpression**. *J Clin Oncol* 2016, **34**(7):721-730.

54. Frampton GM, Ali SM, Rosenzweig M, Chmielecki J, Lu X, Bauer TM, Akimov M, Bufill JA, Lee C, Jentz D *et al*: **Activation of MET via diverse exon 14 splicing alterations occurs in multiple tumor types and confers clinical sensitivity to MET inhibitors**. *Cancer Discov* 2015, **5**(8):850-859.

55. Schrock AB, Frampton GM, Suh J, Chalmers ZR, Rosenzweig M, Erlich RL, Halmos B, Goldman J, Forde P, Leuenberger K *et al*: **Characterization of 298 Patients with Lung Cancer Harboring MET Exon 14 Skipping Alterations**. *J Thorac Oncol* 2016, **11**(9):1493-1502.

56. Lee J, Ou SH, Lee JM, Kim HC, Hong M, Kim SY, Jang J, Ahn S, Kang SY, Lee S *et al*: **Gastrointestinal malignancies harbor actionable MET exon 14 deletions**. *Oncotarget* 2015, **6**(29):28211-28222.

57. Zenali M, deKay J, Liu Z, Hamilton S, Zuo Z, Lu X, Bakkar R, Mills G, Broaddus R: **Retrospective Review of MET Gene Mutations**. *Oncoscience* 2015, **2**(5):533-541.

58. Grabiner BC, Nardi V, Birsoy K, Possemato R, Shen K, Sinha S, Jordan A, Beck AH, Sabatini DM: **A diverse array of cancer-associated MTOR mutations are hyperactivating and can predict rapamycin sensitivity**. *Cancer Discov* 2014, **4**(5):554-563.

59. Kong Y, Si L, Li Y, Wu X, Xu X, Dai J, Tang H, Ma M, Chi Z, Sheng X *et al*: **Analysis of mTOR Gene Aberrations in Melanoma Patients and Evaluation of Their Sensitivity to PI3K-AKT-mTOR Pathway Inhibitors**. *Clin Cancer Res* 2016, **22**(4):1018-1027.

60. Rodrik-Outmezguine VS, Okaniwa M, Yao Z, Novotny CJ, McWhirter C, Banaji A, Won H, Wong W, Berger M, de Stanchina E *et al*: **Overcoming mTOR resistance mutations with a new-generation mTOR inhibitor**. *Nature* 2016, **534**(7606):272-276.

61. Ohashi K, Sequist LV, Arcila ME, Lovly CM, Chen X, Rudin CM, Moran T, Camidge DR, Vnencak-Jones CL, Berry L *et al*: **Characteristics of lung cancers harboring NRAS mutations**. *Clin Cancer Res* 2013, **19**(9):2584-2591.

62. Schirripa M, Cremolini C, Loupakis F, Morvillo M, Bergamo F, Zoratto F, Salvatore L, Antoniotti C, Marmorino F, Sensi E *et al*: **Role of NRAS mutations as prognostic and predictive markers in metastatic colorectal cancer**. *Int J Cancer* 2015, **136**(1):83-90.

63. Boespflug A, Caramel J, Dalle S, Thomas L: **Treatment of NRAS-mutated advanced or metastatic melanoma: rationale, current trials and evidence to date**. *Ther Adv Med Oncol* 2017, **9**(7):481-492.

64. Corless CL, Schroeder A, Griffith D, Town A, McGreevey L, Harrell P, Shiraga S, Bainbridge T, Morich J, Heinrich MC: **PDGFRA mutations in gastrointestinal stromal tumors: frequency, spectrum and in vitro sensitivity to imatinib**. *J Clin Oncol* 2005, **23**(23):5357-5364.

65. Dai J, Kong Y, Si L, Chi Z, Cui C, Sheng X, Mao L, Li S, Lian B, Yang R *et al*: **Large-scale analysis of PDGFRA mutations in melanomas and evaluation of their sensitivity to tyrosine kinase inhibitors imatinib and crenolanib**. *Clin Cancer Res* 2013, **19**(24):6935-6942.

66. Debiec-Rychter M, Cools J, Dumez H, Sciot R, Stul M, Mentens N, Vranckx H, Wasag B, Prenen H, Roesel J *et al*: **Mechanisms of resistance to imatinib mesylate in gastrointestinal stromal tumors and activity of the PKC412 inhibitor against imatinib-resistant mutants**. *Gastroenterology* 2005, **128**(2):270-279.

67. Vogt PK, Kang S, Elsliger MA, Gymnopoulos M: **Cancer-specific mutations in phosphatidylinositol 3-kinase**. *Trends Biochem Sci* 2007, **32**(7):342-349.

68. Wang L, Hu H, Pan Y, Wang R, Li Y, Shen L, Yu Y, Li H, Cai D, Sun Y *et al*: **PIK3CA mutations frequently coexist with EGFR/KRAS mutations in non-small cell lung cancer and suggest poor prognosis in EGFR/KRAS wildtype subgroup**. *PLoS One* 2014, **9**(2):e88291.

69. Gymnopoulos M, Elsliger MA, Vogt PK: **Rare cancer-specific mutations in PIK3CA show gain of function**. *Proc Natl Acad Sci U S A* 2007, **104**(13):5569-5574.

70. Manca A, Lissia A, Capone M, Ascierto PA, Botti G, Caraco C, Stanganelli I, Colombino M, Sini M, Cossu A *et al*: **Activating PIK3CA mutations coexist with BRAF or NRAS mutations in a limited fraction of melanomas**. *J Transl Med* 2015, **13**:37.

71. Ahn SM, Ansari AA, Kim J, Kim D, Chun SM, Kim J, Kim TW, Park I, Yu CS, Jang SJ: **The somatic POLE P286R mutation defines a unique subclass of colorectal cancer featuring hypermutation, representing a potential genomic biomarker for immunotherapy**. *Oncotarget* 2016, **7**(42):68638-68649.

72. Domingo E, Freeman-Mills L, Rayner E, Glaire M, Briggs S, Vermeulen L, Fessler E, Medema JP, Boot A, Morreau H *et al*: **Somatic POLE proofreading domain mutation, immune response, and prognosis in**

colorectal cancer: a retrospective, pooled biomarker study. *Lancet Gastroenterol Hepatol* 2016, **1**(3):207-216.

73. Jin G, Kim MJ, Jeon HS, Choi JE, Kim DS, Lee EB, Cha SI, Yoon GS, Kim CH, Jung TH *et al*: **PTEN mutations and relationship to EGFR, ERBB2, KRAS, and TP53 mutations in non-small cell lung cancers**. *Lung Cancer* 2010, **69**(3):279-283.

74. Lin EI, Tseng LH, Gocke CD, Reil S, Le DT, Azad NS, Eshleman JR: **Mutational profiling of colorectal cancers with microsatellite instability**. *Oncotarget* 2015, **6**(39):42334-42344.

75. Nassif NT, Lobo GP, Wu X, Henderson CJ, Morrison CD, Eng C, Jalaludin B, Segelov E: **PTEN mutations are common in sporadic microsatellite stable colorectal cancer**. *Oncogene* 2004, **23**(2):617-628.

76. Facchinetti F, Loriot Y, Kuo MS, Mahjoubi L, Lacroix L, Planchard D, Besse B, Farace F, Auger N, Remon J *et al*: **Crizotinib-Resistant ROS1 Mutations Reveal a Predictive Kinase Inhibitor Sensitivity Model for ROS1- and ALK-Rearranged Lung Cancers**. *Clin Cancer Res* 2016, **22**(24):5983-5991.

77. Song A, Kim TM, Kim DW, Kim S, Keam B, Lee SH, Heo DS: **Molecular Changes Associated with Acquired Resistance to Crizotinib in ROS1-Rearranged Non-Small Cell Lung Cancer**. *Clin Cancer Res* 2015, **21**(10):2379-2387.

78. Olivier M, Hollstein M, Hainaut P: **TP53 mutations in human cancers: origins, consequences, and clinical use**. *Cold Spring Harb Perspect Biol* 2010, **2**(1):a001008.

79. Suraweera N, Duval A, Reperant M, Vaury C, Furlan D, Leroy K, Seruca R, Iacopetta B, Hamelin R: **Evaluation of tumor microsatellite instability using five quasimonomorphic mononucleotide repeats and pentaplex PCR**. *Gastroenterology* 2002, **123**(6):1804-1811.
